# From post-war reconstruction to the twenty-first century – ophthalmic pathology in Freiburg 1945–2015: review of 39,256 surgical specimens from various topographical regions collected over 71 years at a large German tertiary eye care centre

**DOI:** 10.1186/s12886-025-04056-1

**Published:** 2025-05-07

**Authors:** Mateusz Glegola, Tabea Schulz, Simone Nuessle, Daniel Boehringer, Julia Stifter, Thomas Englbrecht, Thomas Reinhard, Johannes Haedrich, Claudia Auw-Haedrich

**Affiliations:** 1 Eye Center at Medical Center, University of Freiburg, Killianstraße 5, Freiburg, 79106 Germany; 2 Eye Hospital, University Hospital of Basel, Switzerland, Mittlere Str. 91, Basel, 4056 Switzerland

**Keywords:** Ophthalmic pathology, Eyelid, Cornea, Conjunctiva, Eyeball, Temporal artery, Time trends

## Abstract

**Background:**

Ophthalmic pathology at the Eye Center at Medical Center, University of Freiburg, Germany, looks back on a tradition of more than 150 years. Surgical specimens and associated histological diagnoses have been archived since 1945. This study is the first of its size to include 39,256 specimens examined in a single ophthalmic pathology laboratory over 71 years.

**Methods:**

We retrospectively studied ophthalmic pathology reports and clinical records of histological specimens archived between 1945 and 2015 and compared our results with 22 studies from the literature. Samples were grouped by localisation and histopathological diagnoses assigned to various categories. Both were coded and entered into a database together with the year of surgery, patient sex and age at excision.

**Results:**

The patients’ age at surgery was documented in 38,845 cases (99%), of which 19,601 were female (50.5%) and 19,244 were male patients (49.5%). The bimodal frequency distribution of specimens by patient age has a similar shape to that recorded 1941–1995 for Atlanta, USA and 1959–2021 for the Swedish population. Most specimens originated from the eyelid (50%), followed by cornea (16%), conjunctiva (14%), eyeball (9.1%), temporal artery (3.9%) and other locations (6.7%) comprising 16 less frequent topographies. The proportion of eyelid (corneal, conjunctival) lesions significantly increased fourfold (fivefold, twofold) during our study period (each *p* < .001); that of enucleations and temporal artery biopsies decreased significantly 38- and 3.6-fold (each *p* < .001). Concurrently, the numbers of eyelid, corneal, conjunctival and temporal artery specimens have significantly grown (each *p* < .001). Annual sample numbers increased significantly across the various medical directors’ tenures (1945–1967: median = 78; 1968–1987: median = 454; 1988–2002: median = 670; 2003–2015: median = 1,445) (*p* < .001).

**Conclusions:**

Historical events, general population developments and new surgical techniques and treatment options caused changes in the occurrence of various ocular and periocular specimens. Our study data contribute to providing an overall picture of the nature and relative frequency of ocular conditions leading to surgical excision of specimens with subsequent histopathological examination. A continuous sharp increase in case numbers since 1987 clearly exceeds the demographic trend emphasising the ever-growing importance of the sub-speciality of ophthalmic pathology. Ideally, histopathological assessments should be conducted by experienced ophthalmologists with surgical and pathology expertise, or by experienced pathologists with ophthalmology expertise, to ensure optimal patient care.

## Background

Ophthalmic pathologists evaluate specimens excised from the patient’s eye and ocular adnexa to provide the eye surgeon or treating ophthalmologist with an accurate diagnosis. Ophthalmic pathology is therefore essential for day-to-day clinicopathological correlations and ophthalmological research [1–5]. For generations, the ophthalmic pathology laboratory was the hub of teaching and expertise in the field of ophthalmology at every German university hospital. Increasing specialisation in neuro-ophthalmologists, eyelid specialists, strabologists, paediatric ophthalmologists, anterior segment surgeons and conservative and surgical retinologists makes the ophthalmic pathologist working in all areas of the eye and ocular adnexa a kind of “universal scholar” [5].

Ophthalmic pathology at the Eye Center at Medical Center of the University of Freiburg, Germany looks back on a history of more than 150 years. It is a sub-speciality rich in tradition, with a lineage of great ophthalmologists such as Carl-Joseph Beck (1794–1838), Wilhelm Manz (1833–1911), Theodor Axenfeld (1867–1930), Wilhelm Wegner (1898–1972), Hanns-Hellmut Unger (1919–2008) und Heinrich Witschel (1937–2019). Each of these luminaries made significant contributions to the further development and flourishing of ophthalmic pathology and to the general progress of ophthalmology up to the present day [6, 7]. But even with its thriving history, the Freiburg Eye Hospital was not spared from the events of the Second World War.

**Fig. 1:**
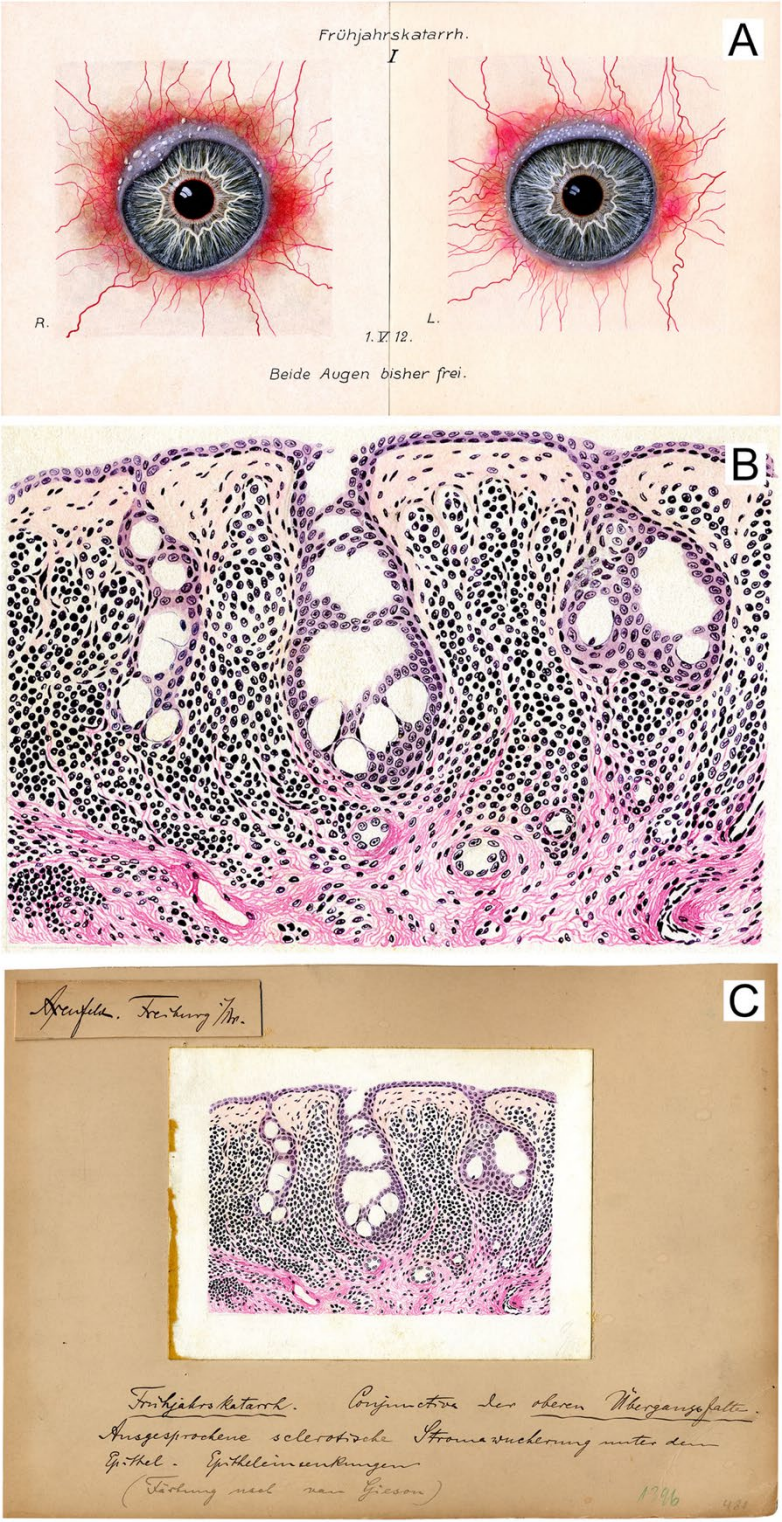
Clinicopathological correlation from 1912*.* Hand-made illustration from our archive, drawn by Berta Axenfeld, Theodor Axenfeld’s wife, showing the clinical (A) and histological (B) appearance of an untreated conjunctivitis vernalis (spring catarrh) of 1 May 1912, labelled with handwritten notes by Theodor Axenfeld and bearing his signature (C). Axenfeld’s notes translate as follows: “Axenfeld. Freiburg/Br.” (above), “Spring catarrh. Conjunctiva of the upper fornix. Pronounced sclerotic stromal proliferation under the epithelium. Epithelial depressions. (Staining according to van Gieson)“ (bottom)

The surprise air raid by the Royal Air Force on the evening of November 1944 was disastrous for the entire Freiburg University Hospital. It also meant a deep cut for ophthalmic pathology activities. While a provisional eye clinic had been prepared in the “Wonnhalde” on the premises of the “Rebhaus” Sanatorium outside the city as a precautionary measure, and these facilities were swiftly occupied within a few days after the raid, the histology laboratory continued to operate fully at the original eye hospital site until the very last moment. Unfortunately, large areas of Freiburg, including the eye clinic, were completely destroyed, as was the histology lab, leading to the total loss of all samples and records prior to 1945. All records, except for a few hand-made illustrations, some of which had skilfully been drawn by Berta Axenfeld, Theodor Axenfeld’s wife. As an example of a clinicopathological correlation from that time, Fig. 1 shows the clinical and histological appearance of an untreated conjunctivitis vernalis (spring catarrh) on 1 May 1912, labelled with handwritten notes by Theodor Axenfeld and bearing his signature.

Were it not for Franz Jankovsky, since 1938 caretaker of the eye clinic and also its porter, who salvaged ophthalmological and histological utensils and instruments from the ruins and successfully repaired them, the histology laboratory’s equipment would also have been completely lost. Despite having no formal training, he had developed into one of the most skilful laboratory technicians since 1939. His accuracy in histological diagnostics was verified by Wilhelm Wegner himself, and he became the (co-)author of various scientific papers introducing new embedding methods, histological sectioning and staining techniques [6]. It was also he who laid the foundation for a new collection of ophthalmopathological specimens with the first sample dated 13 February 1945.

More than 70 years later, we decided to review this sample archive from our histopathological laboratory, which has been known as the Specialised Ophthalmic Pathology Laboratory since 2019. From 2016 onwards, two doctoral theses were initiated, under which all histological specimens in the archive, along with their associated findings, were reviewed, histologically re-evaluated, entered into a database, and analysed. Our goal was to assess how the eventful history of the Freiburg Eye Center, along with the development of its resources and surgical procedures on the one hand, and the progression of diseases and injuries in the population of its catchment area on the other, might have influenced the nature and frequency of submitted specimens and established histopathological diagnoses.

Various studies have analysed histologically examined surgical samples, evaluating time-dependent changes based on selected topographical areas, diagnostic categories, individual years, or specific time periods. To our knowledge, the present study is the first of its size to include almost 40,000 specimens from a single ophthalmic pathology laboratory over a 71-year time span (1945–2015), mapping changes in the range of ocular and periocular anatomical origins, as well as in the spectrum of diagnostic categories of surgically obtained specimens.

## Methods

### Specimens

Our ophthalmic pathology laboratory prepares and examines histological samples which are primarily excisional and incisional biopsies of lesions as well as surgical resection specimens, submitted mainly from our own eye clinic. Since the mid-1990s, we also receive samples from hospitals, pathological institutes and practising ophthalmologists throughout Germany and beyond. All samples were processed according to standard procedures, some underwent special staining. The specimens were examined by light microscopy and some by transmission electron microscopy. Diagnoses were made by experienced ophthalmic pathologists, who are also eye surgeons, applying state-of-the-art histopathological criteria according to the respective topographical regions. In addition, a small number of samples were examined consultatively at the Institute for Surgical Pathology and at the Histology Laboratory of the Clinic for Dermatology and Venereology, both at the University Medical Center Freiburg.

### Our study

We retrospectively studied ophthalmic pathology reports with clinical data of all histological samples archived at the Eye Center at Medical Center, University of Freiburg between 1945 and 2015. The specimens had been excised *in domo* during surgeries; we categorised them according to topographical regions (eyelid, cornea, conjunctiva, eyeball, temporal artery, and a combined group “other location”) for inclusion in our study. Diagnoses were assigned to various categories such as inflammation, degeneration, scar tissue, ulcer or tumour. If required or useful, further subdivisions were made, e.g. in the case of corneal dystrophies into the individual underlying types of dystrophy (i.e. Fuchs’, macular, lattice, or granular dystrophy). Some specimens were assigned to more than one single diagnosis, e.g. “benign tumour” as well as “inflammation”, resulting in more diagnoses than collected samples. Malignant tumours were classified based on existing metastatic potential (EMP), absent metastatic potential (AMP) and on their cell of origin. Additional information such as patient sex and age at surgery was also collected. In rare cases, when the diagnosis was not entirely definite, further histological examinations and, if necessary, consultations were held on a national or international level.

Statistical analyses of our data were performed using R-Studio [8, 9]. By convention, *p*-values (two-sided) below .05 were considered statistically significant. We used the Shapiro–Wilk test [10] to analyse if data deviated significantly from a normal distribution. Levene’s test [11] was utilised to assess the equality of variances (homoscedasticity) between two or more groups. Statistical comparisons between groups were performed to assess changes in the relative frequency of samples during the study period. A one-way analysis of variance (ANOVA) was applied if the data were normally distributed and the variances were homogeneous. The Kruskal–Wallis non-parametric test [12] was used for non-normally distributed data. Post-hoc pairwise comparisons were evaluated at a significance level of *p* < .05 without adjustment for multiple testing, in order not to overlook potentially interesting results by increasing the probability of a type II error, i.e. not rejecting the null hypothesis (*H*_0_) even though it is actually false. ANOVA was followed by Tukey’s Honest Significant Difference (HSD) test [13]; Dunn’s post-hoc test on ranks [14] was performed after the Kruskal–Wallis test [12]. Hartigans’ dip test for unimodality [15] was used to measure multimodality in the frequency distribution of specimens by patient age at surgery.

### Studies for comparison

Several studies involving ophthalmic pathology specimens from various topographical areas were used for comparison with the number and frequency of eyelid, corneal, conjunctival, eyeball and temporal artery diagnoses in the specimens collected in our archive. To facilitate comparability, we regrouped benign and malignant tumours of the eyelid and conjunctiva, as well as corneal dystrophies, in some of these studies to readjust their categorisations.

Rohrbach et al. [16] reviewed the histological diagnoses on 1,835 specimens collected in 1900, 1920, 1940, 1960, 1980 and 1990 in the ophthalmic pathology laboratory at the University Eye Hospital Tübingen, Germany, covering the entire spectrum of ocular and periocular changes. Spraul and Grossniklaus [17] analysed 24,444 surgical specimens from the L.F. Montgomery Ophthalmic Pathologic Laboratory, Emory University, Atlanta, GA, USA, collected from 1941 to 1995 and covering the whole spectrum of ocular and periocular lesions. Changes in the relative frequency of topographical regions were examined by the authors by comparing data from three time periods (1941–1945, 1970 and 1995).

Aurora and Blodi [18] evaluated 892 eyelid tumours collected 1932–1969 at the Department of Ophthalmology, University of Iowa, College of Medicine, Iowa City, IA, USA. Welch and Duke [19] reviewed 617 eyelid tumours collected from 1952 to 1956, many of which were studied in the ophthalmic pathology laboratory at the Wilmer Ophthalmological Institute of The Johns Hopkins University and Hospital, Baltimore, MD, USA. Font et al. [20] analysed 1,474 eyelid lesions examined histologically in the years 1970 to 2000 at the Doheny Eye Institute, Pasadena, CA, USA. Tesluk [21] examined 720 eyelid tumours collected between 1980 and 1982 at Wills Ophthalmic Pathology Laboratory, Wills Eye Hospital, Thomas Jefferson University, Philadelphia, PA, USA. Deprez and Uffer [22] evaluated 5504 eyelid tumours collected between 1989 and 2007 in the Laboratory of Ophthalmopathology of the Hopital Ophtalmique Jules Gonin, Lausanne, Switzerland. Domingo et al. [23] reviewed 1551 tumours comprising 530 from the eyelids, 254 from the conjunctiva, 394 from intraocular locations and 373 from the orbit at the Ocular Pathology Section of the Philippine Eye Research Institute, Manila, Philippines, archived in the period 2003 to 2012.

The Australian Corneal Graft Registry (ACGR) has since 1987 collected information on more than 40,000 corneal grafts including various corneal dystrophies. Keane et al. [24] analysed all corneal grafts performed until the end of 2020 and registered with the ACGR. Musch et al. [25] evaluated 27,372 individuals diagnosed with corneal dystrophy between 2001 and 2007, collected in a US database containing detailed records of all the insured in a national managed-care network throughout the United States. Is not known which of these diagnoses submitted by ophthalmologists or optometrists were confirmed by ophthalmic pathology.

Grossniklaus et al. [26] reviewed and histopathologically classified 2455 conjunctival lesions in adults examined in the Eye Pathology Laboratory of the Wilmer Ophthalmological Institute in Baltimore, MD, USA during a 61-year period from 1923 to 1984. Shields et al. [27] examined a large spectrum of conjunctival tumours comprising 5002 cases from 1974 to 2015 at the Ocular Oncology Service, Wills Eye Hospital, Thomas Jefferson University, Philadelphia, PA, USA.

Hansen et al. [28] assessed the pathology reports of 1,028 bulbar eviscerations and enucleations from three two-year periods 1975–76, 1985–86 and 1995–96, collected by the Eye Pathology Institute, University of Copenhagen, Copenhagen, Denmark. De Gottrau et al. [29] reviewed clinicopathological data and paraffin sections of 1,146 enucleated eyes at the Laboratory of Ophthalmic Pathology of the Department of Ophthalmology, University of Erlangen-Nürnberg, Germany, between 1980 and 1990. Saeed et al. [30] performed a retrospective review of 285 enucleation/evisceration histopathology reports at the Department of Ophthalmology, General Infirmary, Leeds, UK, from 1984 to 2003. Geirsdottir et al. [31] analysed medical records of 56 patients at The National University Hospital of Iceland, Reykjavik, Iceland, who underwent enucleation between 1992 and 2004. Chan et al. [32] assessed patient demographics, clinical indications and pathologic causes of 713 surgically removed eyes received from the University of Toronto Ophthalmic Pathology Laboratory, enucleated between 2004 and 2013 at 28 health centres in Ontario, Canada.

McDonnell et al. [33] reviewed clinical and laboratory findings at the Eye Pathology Laboratory of the Wilmer Ophthalmological Institute at The Johns Hopkins Medical Institutions, Baltimore, MD, USA, of 237 consecutive patients who had 250 temporal artery biopsies performed during a 15-year period (1968–1983). Oh et al. [34] performed a retrospective case–control study of a consecutive cohort of 545 patients who had undergone temporal artery biopsies across five hospitals in NSW, Australia, from 1992 to 2015; specimens were examined at the Royal North Shore Hospital, Sydney, NSW, Australia. Weis et al. [35] studied the clinical records from 119 consecutive patients referred to the Ophthalmology Services in Edmonton, Calgary, Quebec and Ottawa (Canada) for temporal artery biopsy from 2005 to 2010. De la Torre et al. [36] retrospectively reviewed 63 consecutive patients who had undergone temporal artery biopsies at the CEMIC University Hospital in Buenos Aires, Argentina, over an 11-year period 2005–2016. Yuksel et al. [37] studied the medical records, laboratory findings and postoperative complications of 42 consecutive patients operated for temporal artery biopsy at Trakya University, Faculty of Medicine, Edirne, Turkey, from 2011 to 2016.

## Results

### Specimen numbers

**Fig. 2:**
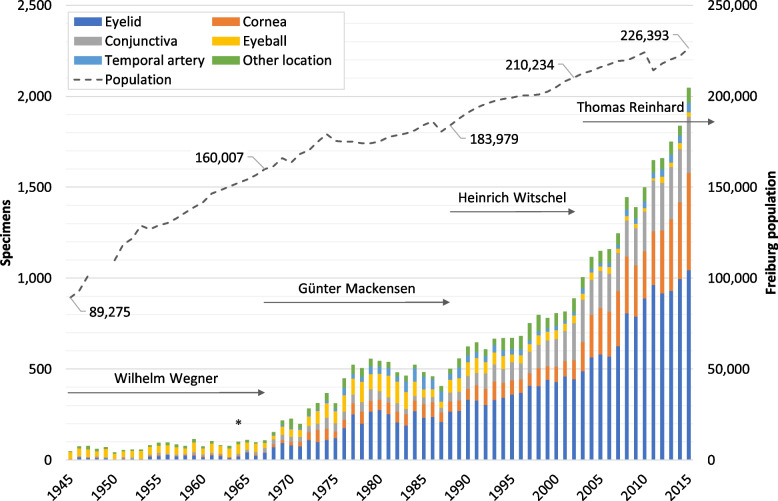
Ophthalmopathological specimens examined 1945–2015. Changes in the number of specimens (*n* = 39,256) from main topographical regions examined during the study period (1945–2015); included are specimens submitted from external medical institutions and practising ophthalmologists since the mid-1990s. Other location: see Fig. 8. Duration of the respective medical directorship (→): Wegner 1945–1967, Mackensen 1968–1987, Witschel 1988–2002, Reinhard 2003–2015; commissioning of the new Freiburg Eye Center in 1964 (*); demographical development in Freiburg city (--): number of inhabitants; no data available 1948–49

During the 71-year study period, a total of 39,256 surgical specimens with 43,169 associated histopathological diagnoses were collected in our archive. An overview on changes in the number of samples from main topographical areas across the observation period is given in Fig. 2. Included are external specimens submitted from eye hospitals, pathology laboratories and practising ophthalmologists since the mid-1990s. Also shown is the demographical development in Freiburg and the respective terms of office of the medical directors of the Freiburg Eye Center. While only 47 to 77 samples were received annually for histological examination in the late 1940s, their number has steadily increased since 1968 to 556 in 1979 (not explicitly shown). After a moderate decline to 405 specimens in 1987, sample frequency increased again to 889 in 2002 and then more rapidly to 2046 specimens in 2015 (not explicitly shown).

**Fig. 3:**
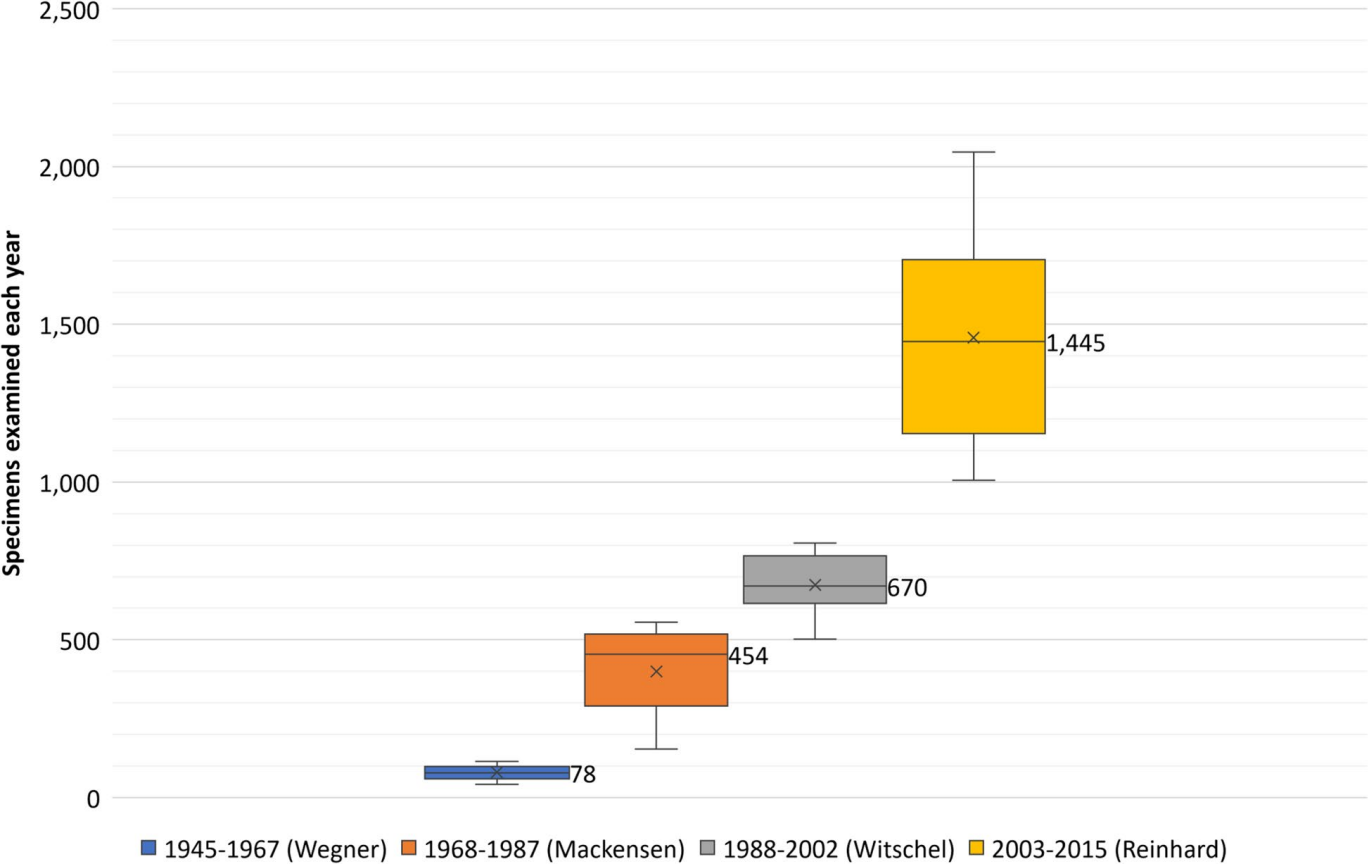
Ophthalmopathological specimens examined annually during four medical directorships. Box-whisker plots of the numbers of specimens examined each year, categorised into four groups according to the respective medical director’s tenure: 1945–1967 (Wegner; total: *n* = 1840), 1968–1987 (Mackensen; total: *n* = 7999), 1988–2002 (Witschel; total: *n* = 10,468) and 2003–2015 (Reinhard; total: *n* = 18,949). Minimum, first quartile [Q1], median (shown), third quartile [Q3], maximum and mean (x) of the annual number of specimens are for 1945–1967: 42, 60, 78, 98, 115 and 80; for 1968–1987: 154, 290, 454, 518.25, 556 and 400; for 1988–2002: 502, 615.5, 670, 766.5, 807 and 674.1; and for 2003–2015: 1005, 1154, 1445, 1704.5, 2046 and 1457.6. The annual number of specimens differed significantly across the various groups (*p* <.001)

According to the respective medical directors’ tenures, annual sample numbers were categorised into four groups (*Mdn*: median of specimens p.a., *n*: total number of specimens in each group): group 1 (1945–1967, Wegner: *Mdn* = 78, *n* = 1,840), group 2 (1968–1987, Mackensen: *Mdn* = 454, *n* = 7999), group 3 (1988–2002, Witschel: *Mdn* = 670, *n* = 10,468) and group 4 (2003–2015, Reinhard: *Mdn* = 1445, *n* = 18,949). The distribution of the annual numbers of specimens within each tenure is represented as box-whisker-plots in Fig. 3. See the caption to Fig. 3 for the minimum, first quartile [Q1], median, third quartile [Q3], maximum and mean values. The Shapiro–Wilk test did not show a significant departure from normality in group 1: *W*(23) = 0.97, *p* = .611, group 3: *W*(15) = 0.97, *p* = .884, and in group 4: *W*(13) = 0.96, *p* = .764, while a significant departure from normality was detected in group 2: *W*(20) = 0.9, *p* = .041. Levene’s test for equality of variances was found to be violated for the present analysis, *F*(3,67) = 19.95, *p* < .001. The effect size, calculated as eta squared (*η*^2^), was 0.47, indicating a large effect. Tukey’s HSD test showed that the variances of the following pairs of groups (gr) are significantly different: gr1-gr2 (*p* = .009), gr1-gr4 (*p* < .001), gr2-gr4 (*p* < .001), gr3-gr4 (*p* < .001). The Kruskal–Wallis test indicated that there is a significant difference in the annual number of specimens between the various medical director’s tenures, *χ*^2^(3) = 64.50, *p* < .001. The effect size (*η*^2^) was 0.92, indicating a large effect. Dunn’s post-hoc test indicated that the mean ranks of the following pairs are significantly different: gr1-gr2 (*p* < .001), gr1-gr3 (*p* < .001), gr1-gr4 (*p* < .001), gr2-gr3 (*p* = .017), gr2-gr4 (*p* < .001).

**Fig. 4:**
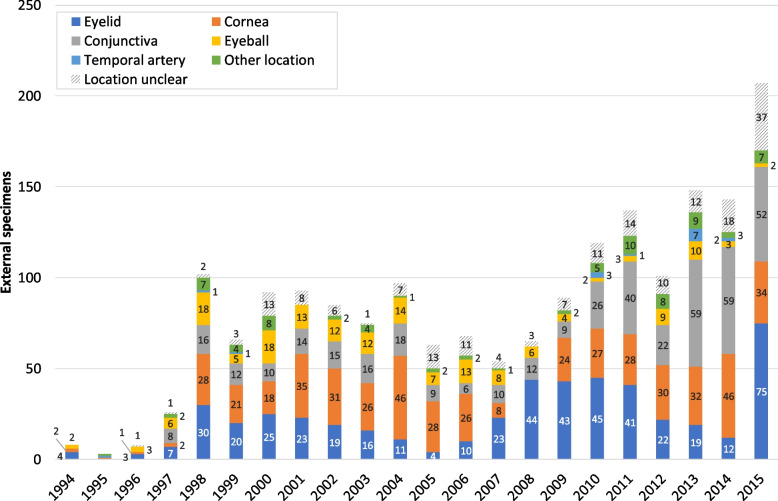
Ophthalmopathological specimens from external submitters 1994–2015. Changes in the number of specimens from main topographical regions submitted from external medical institutions and practising ophthalmologists (1994–2015). Other location: see Fig. 8

Changes in the number of external specimens sent to our ophthalmic pathology laboratory for examination are shown in Fig. 4. Between 1994 and 2015, a total of 1849 external samples were submitted, only 3 to 26 of which were received annually between 1994 and 1997. Since 1998, this number varied between 54 and 148, finally amounting to 207 external specimens (10% of all samples) in 2015.

### Patient age and sex

**Fig. 5:**
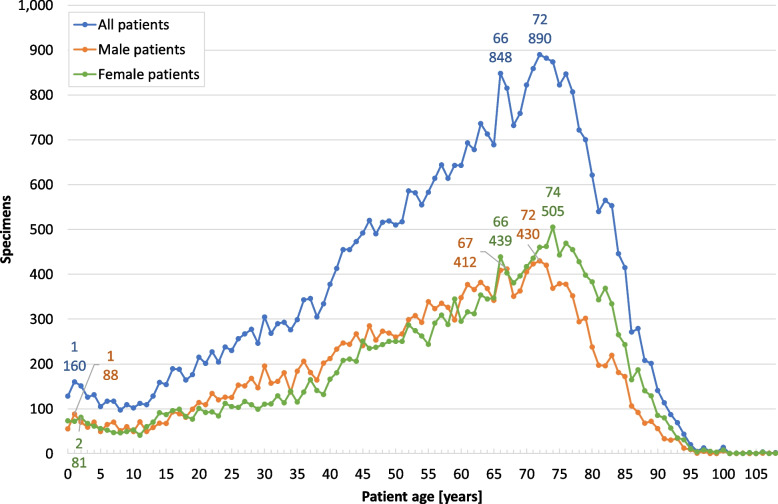
Number of specimens by patient age. Frequency of specimens by age of all patients (*n* = 38,855; range: 0–108 years; median: 61 years), of female (*n* = 19,607; range: 0–108 years; median: 63 years) and of male patients (*n* = 19,248; range: 0–108 years; median: 58 years) at diagnosis during the study period (1945–2015). The three left-skewed distributions (all, f, m) each are non-normal (Shapiro–Wilk test: *p* <.001) and bimodal (Hartigans’ dip test: *p* <.001), with major modes at 72 (all), 74 (f) and 72 years (m) and minor modes at 1 year (all), 2 years (f) and 1 year (m)

The patient age at surgery was documented in 38,855 cases (99%; range: 0–108 years, median: 61 years). Of these, 19,607 (50.5%) were female (f) patients (range: 0–108 years, median: 63 years) and 19,248 (49.5%) were male (m) patients (range: 0–108 years, median: 58 years). The Shapiro–Wilk test showed that frequency distributions of specimens by recorded patient age at surgery, depicted in Fig. 5, depart significantly from a normal distribution each for women, men and all patients combined (f: *W* = 0.93697, *p* < .001; m: *W* = 0.9627, *p* < .001; all: *W* = 0.95756 *p* < .001) showing an asymmetrical left-skewed shape. Hartigan’s dip test rejected unimodality in the frequency distributions for women, men, and all patients respectively significantly indicating multimodality (f: *D* = 0.011964, *p* < .001; m: *D* = 0.011351, *p* < .001; all: *D* = 0.011328 *p* < .001); visual analysis suggests a bimodal distribution in all cases. For all patients combined, the major mode representing the most frequently observed age is 72 years (*n* = 890), while a minor mode occurs at age 1 (*n* = 160) suggesting a bimodal distribution. We also see a distinct shoulder peak at 66 years (*n* = 848). In female and in male patients, the major mode is 74 (*n* = 505) and 72 years (*n* = 430), respectively, with a minor mode at 2 years (*n* = 81) and 1 year (*n* = 88), respectively. A prominent shoulder peak exists for female patients at 66 years of age (*n* = 439; 65 y: *n* = 347, 67 y: *n* = 403) (Fig. 5), the counterpart of which is not really pronounced in 67-year-old male patients (*n* = 412), with almost as many diagnoses at the neighbouring ages of 66 years (*n* = 409) and 68 years (*n* = 351). Table 1 provides an overview of the most frequent “Top 20” diagnostic categories of histologically examined surgical specimens from female patients age 66 and 67 years combined, and at age 74 years. Table 2 contains the respective “Top 20” diagnostic categories of specimens from male patients age 66 and 67 years combined, and at age 72 years.

**Table 1:** Most frequent diagnostic categories in female patients aged 66/67 and 74 years

Female patients, 66 and 67 years of age			Female patients, 74 years of age		
Diagnostic category	n	(%)	Diagnostic category	n	(%)
Benign eyelid tumour	196	23	Benign eyelid tumour	94	19
Malignant AMP eyelid tumour	101	12	Malignant AMP eyelid tumour	87	17
Inflammatory eyelid lesion (mostly chalazion)	62	7.4	Corneal dystrophy (mostly Fuchs’ dystrophy except in 3 cases)	48	10
Normal eyelid tissue (mostly re-resection specimens)	53	6.3	Inflammatory eyelid lesion (mostly chalazion)	31	6.1
Corneal dystrophy (mostly Fuchs’ dystrophy except in 3 cases)	45	5.3	Normal eyelid tissue (mostly re-resection specimens)	30	5.9
Pterygium and pinguecula	44	5.2	Degenerative corneal change	24	4.8
Degenerative corneal change	40	4.8	Inflamed temporal artery	23	4.6
Normal temporal artery	23	2.7	Pterygium and pinguecula	22	4.4
Inflamed temporal artery	22	2.6	Degenerative temporal artery	16	3.2
Benign conjunctival tumour	20	2.4	Benign conjunctival tumour	12	2.4
Degenerative temporal artery	20	2.4	Corneal scarring	10	2.0
Malignant EMP eyeball tumour	19	2.3	Inflammatory conjunctival lesion	8	1.6
Malignant AMP conjunctival tumour	14	1.7	Degenerative eyelid lesion	8	1.6
Malignant EMP eyelid tumour	12	1.4	Malignant EMP eyeball tumour	7	1.4
Inflammatory conjunctival lesion	11	1.3	Vascular disease associated enucleation	5	1.0
Corneal scarring	11	1.3	Degenerative lens change (cataract)	5	1.0
Inflammatory corneal lesion	8	1.0	Cataract surgery associated enucleation	4	0.79
Glaucoma associated enucleation	8	1.0	Malignant EMP eyelid tumour	4	0.79
Eyelid scarring	7	0.83	Normal temporal artery	4	0.79
Malignant EMP orbital tumour	7	0.83	Malignant EMP conjunctival tumour	3	0.59
Sum^a^	723	86	Sum^a^	445	88
Total number of diagnoses	842		Total number of diagnoses	505	

Relative frequency of the 20 most common diagnostic categories in all female patients aged 66 (*n* = 439) and 67 years (*n* = 403), and aged 74 years (*n* = 505) respectively, at the time of surgery during the observation period (1945–2015). AMP: absent metastatic potential, EMP: existing metastatic potential

^a^Sum of diagnoses in female patients aged 66 and 67 years, or 74 years respectively, within the “Top 20”

**Table 2:** Most frequent diagnostic categories in male patients aged 66/67 and 72 years

Male patients, 66 and 67 years of age			Male patients, 72 years of age		
Diagnostic category	n	(%)	Diagnostic category	n	(%)
Benign eyelid tumour	183	22	Benign eyelid tumour	71	17
Malignant AMP eyelid tumour	88	11	Malignant AMP eyelid tumour	53	12
Inflammatory eyelid lesion (mostly chalazion)	75	9.1	Pterygium and pinguecula	43	10
Pterygium and pinguecula	60	7.3	Inflammatory eyelid lesion (mostly chalazion)	37	8.6
Normal eyelid tissue (mostly re-resection specimens)	45	5.5	Degenerative corneal change	23	5.3
Corneal dystrophy (all Fuchs’ dystrophy)	38	4.6	Corneal dystrophy (all Fuchs’ dystrophy)	23	5.3
Degenerative corneal change	34	4.1	Normal eyelid tissue (mostly re-resection specimens)	21	4.9
Malignant EMP eyeball tumour	19	2.3	Benign conjunctival tumour	14	3.3
Trauma associated enucleation	19	2.3	Degenerative temporal artery	12	2.8
Degenerative temporal artery	18	2.2	Malignant AMP conjunctival tumour	8	1.9
Benign conjunctival tumour	17	2.1	Normal temporal artery	7	1.6
Inflammatory corneal lesion	13	1.6	Trauma associated enucleation	7	1.6
Degenerative eyelid lesion	13	1.6	Inflammatory conjunctival lesion	6	1.4
Corneal scarring	12	1.5	Corneal scarring	6	1.4
Malignant EMP eyelid tumour	12	1.5	Degenerative eyelid lesion	6	1.4
Normal temporal artery	11	1.3	Malignant EMP eyelid tumour	6	1.4
Inflammatory conjunctival lesion	9	1.1	Malignant EMP eyeball tumour	6	1.4
Malignant EMP conjunctival tumour	9	1.1	Eyelid scarring	5	1.2
Glaucoma associated enucleation	8	1.0	Inflamed temporal artery	5	1.2
Corneal ulcer or keratitis associated enucleation	8	1.0	Normal conjunctiva	4	0.93
Sum^a^	691	84	Sum^a^	363	84
Total number of diagnoses	821		Total number of diagnoses	430	

Relative frequency of the 20 most common diagnostic categories in all male patients aged 66 (*n* = 409) and 67 years (*n* = 412), and aged 72 years (*n* = 430) respectively, at the time of surgery during the observation period (1945–2015). AMP: absent metastatic potential, EMP: existing metastatic potential

^a^Sum of diagnoses in male patients aged 66 and 67 years, or 72 years respectively, within the “Top 20”

**Fig. 6:**
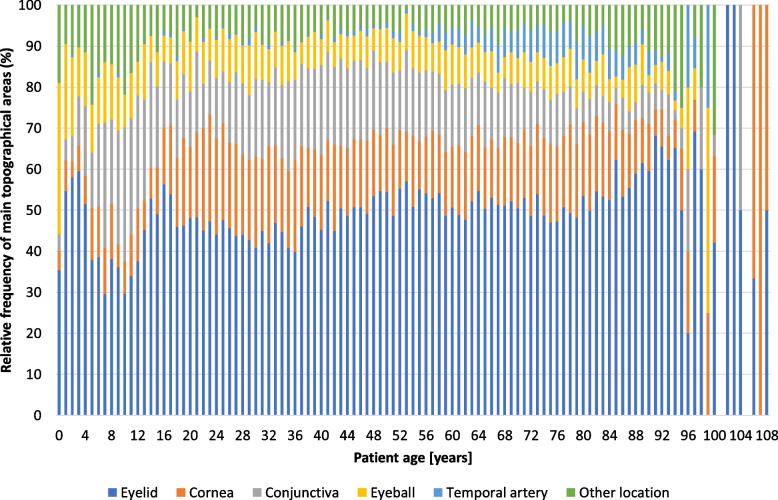
Topographical regions by patient age at surgery. Relative frequency of main topographical areas by patient age at surgery during the study period (1945–2015). Other location: see Fig. 8

Figure 6 shows the relative frequency of the five major topographical areas and of the summarised group “other location” by patient age at surgery across the study period (1945–2015). In most patient ages, about half of the specimens were taken from the eyelid (range: 40–57%) with this proportion increasing to about two thirds (68%) above the age of 80. Only in patients aged a few months (age “0”) and aged 5–12 years was the proportion of eyelid samples markedly lower at 30–39%, “in favour” of eyeball specimens (enucleations) at age “0” years (37%) and a larger proportion of conjunctival lesions at the age of 6–12 years (20–33%). Major inconsistencies can be observed in the percentages of eyelid specimens beyond a patient age of 95 years. Corneal samples accounted for only 4–8% of all specimens at patient age 0–4 years, for 6–17% up to the age of 18 years, they increased to 14–26% at the age of 19–42 years and dropped again to 11–22% at 43–90 years. Proportions were not consistent in patients above 90 years of age. The relative frequency of conjunctival samples was less than 4% in patients below 1 year of age at the time of surgery, it increased to 17% at 1–5 years of age, was distinctly elevated at age 6–15 years (20–33%) and then remained between 10 and 22% up to 77 years of age (Fig. 6). Eyeballs were most frequent at 37% during the first few months after birth (age “0”). Their fraction dropped to 19% until the age of 2 years, decreased further to 11–15% up to an age of 9 years, then generally remained between 5 and 13% until 97 years of age. Specimens from the temporal artery were rarely excised before 16 years of age. Up to an age of 50 years, their relative frequency was less than 2%. Thereafter, however, temporal artery excisions became more frequent until a proportion of 9.1% was reached at the age of 81, then they steadily dropped to 2.3% for patients aged 94. The percentage of samples taken at other locations generally remained below 12% in patients aged 13 to 93 years, while their proportion was up to 24% among the under-13s and as high as 32% for those aged 93 and over (Fig. 6).

### Topographical regions

**Fig. 7:**
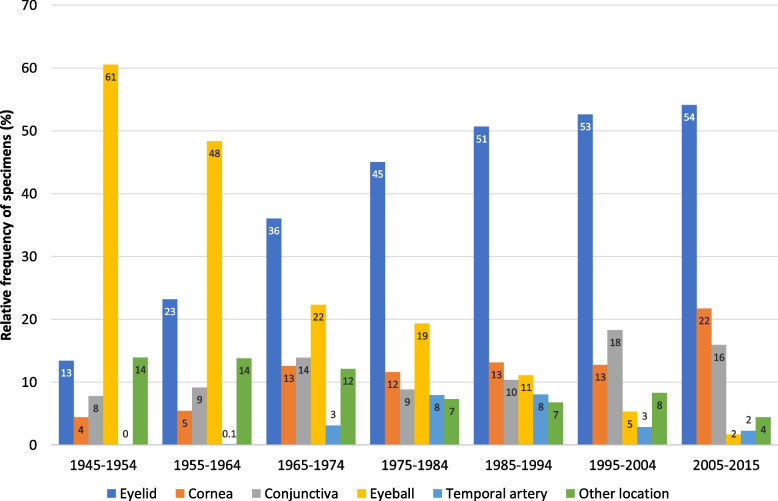
Changes in the relative frequency of specimens from main topographical regions. Relative frequency of samples from the main topographical areas received for histological examination within the 7 intervals of the study period (1945–2015). Other location: see Fig. 8. See text for statistical comparisons between the intervals

Changes in the number and/or relative frequency of specimens obtained from the most important topographical regions and examined between 1945 and 2015 are shown in Fig. 7 and in Table 3. Intervals 1–6 were set at 10 years (1945–1954, 1955–1964, 1965–1974, 1975–1984, 1985–1994, 1995–2004), with the exception of interval 7 covering 11 years (2005–2015). The Kruskal–Wallis test followed by Dunn’s post-hoc test indicated a significant increase in the annual specimen numbers across our study period, expressed as median values within the respective interval: from 8 eyelid samples p.a. in 1945–1954 to 888 p.a. in 2005–2015 (*χ*^2^(6) = 67.49, *p* < .001), from 2.5 to 302 corneal excisions (*χ*^2^(6) = 62.06, *p* < .001), and from 5 to 220 conjunctival specimens p.a. (*χ*^2^(6) = 64.38, *p* < .001). The annual number of enucleated eyeballs increased significantly from 39 in 1945–1954 to 92 in 1975–1974, then significantly decreased to 24 p.a. in 2005–2015 (*χ*^2^(6) = 47.25, *p* < .001). Temporal artery biopsies increased significantly from 8 samples p.a. in 1965–1974 to 40 p.a. in 1985–1994, followed by a significant decrease to 21.5 specimens p.a. in 1995–2004 and yet another, but not significant, rise to 35 annual biopsies in 2005–2015 (*χ*^2^(4) = 32.59, *p* < .001).

**Table 3:** Changes in specimens from main topographical regions

Time period	1945–1954	1955–1964	1965–1974	1975–1984	1985–1994	1995–2004	2005–2015	Total number
Eyelid	83 (13%)	210 (23%)	746 (36%)	2,206 (45%)	2,847 (51%)	4,374 (53%)	9,107 (54%)	19,573 (50%)
Cornea	27 (4.4%)	49 (5.4%)	260 (13%)	568 (12%)	738 (13%)	1,061 (13%)	3,656 (22%)	6,359 (16%)
Conjunctiva	48 (7.8%)	83 (9.2%)	287 (14%)	431 (8.8%)	582 (10%)	1,519 (18%)	2,675 (16%)	5,625 (14%)
Eyeball	374 (61%)	438 (48%)	462 (22%)	945 (19%)	624 (11%)	438 (5.3%)	274 (1.6%)	3,555 (9.1%)
Temporal artery	0 (0%)	1 (0.1%)	64 (3.1%)	389 (7.9%)	451 (8.0%)	235 (2.8%)	377 (2.2%)	1,517 (3.9%)
Other location	86 (14%)	125 (14%)	251 (12%)	357 (7.3%)	380 (6.8%)	689 (8.3%)	739 (4.4%)	2,627 (6.7%)
Sum	618	906	2,070	4,896	5,622	8,316	16,828	39,256

**Fig. 8:**
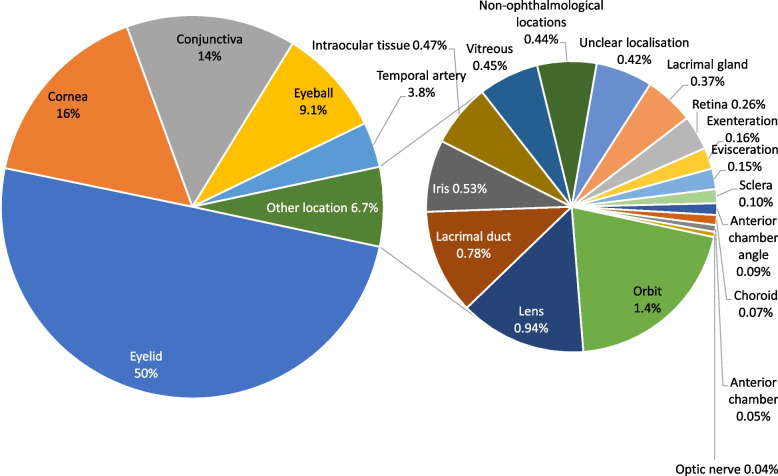
Relative frequency of specimens from different topographical regions. Relative frequency of surgical specimens from various topographical areas received during the study period (1945–2015). The %-values given for the individual components of “other location” refer to the total of all localisations

Number and relative frequency of specimens from main topographical areas received within 10-year intervals (2005–2015: 11 years) during the observation period (1945–2015). Other location: orbit, lens, lacrimal duct, iris, vitreous, lacrimal gland, retina, exenteration, choroid, evisceration, sclera, optic nerve and further sites, see chapter “Results” and Fig. 8. Percentages may not total 100 due to rounding

Over the various medical directors’ tenures, we analysed the development of the annual sample numbers from those topographical areas where these numbers have steadily increased over time, namely the eyelid, cornea and conjunctiva. The following groups resulted for *eyelid* specimens: gr 1 (1945–1967, Wegner: *Mdn* = 19, *n* = 398), gr 2 (1968–1987, Mackensen: *Mdn* = 195, *n* = 3526), gr 3 (1988–2002, Witschel: *Mdn* = 360, *n* = 5488) and gr 4 (2003–2015, Reinhard: *Mdn* = 807, *n* = 10,161). The Kruskal–Wallis test revealed a significant difference in the annual number of eyelid specimens between the four tenures, *χ*^2^(3) = 64.71, *p* < .001. The effect size (*η*^2^) was 0.92, indicating a large effect. Dunn’s post-hoc test indicated that the mean ranks of the following pairs are significantly different: gr1-gr2 (*p* < .001), gr1-gr3 (*p* < .001), gr1-gr4 (*p* < .001), gr2-gr3 (*p* = .015), gr2-gr4 (*p* < .001).

For *corneal* samples, the groups were: gr 1 (1945–1967, Wegner: *Mdn* = 4, *n* = 88), gr 2 (1968–1987, Mackensen: *Mdn* = 59, *n* = 1024), gr 3 (1988–2002, Witschel: *Mdn* = 82, *n* = 1197) and gr 4 (2003–2015, Reinhard: *Mdn* = 297, *n* = 4050). The Kruskal–Wallis test showed that the annual number of corneal specimens between the various tenures are significantly different, *χ*^2^(3) = 62.35, *p* < .001. The effect size (*η*^2^) was 0.89, indicating a large effect. Dunn’s post-hoc test indicated a significant difference in the mean ranks of the following pairs: gr1-gr2 (*p* < .001), gr1-gr3 (*p* < .001), gr1-gr4 (*p* < .001), gr2-gr4 (*p* < .001), gr3-gr4 (*p* = .035).

For *conjunctival* samples, the following groups resulted: gr 1 (1945–1967, Wegner: *Mdn* = 7, *n* = 180), gr 2 (1968–1987, Mackensen: *Mdn* = 34, *n* = 760), gr 3 (1988–2002, Witschel: *Mdn* = 95, *n* = 1587) and gr 4 (2003–2015, Reinhard: *Mdn* = 220, *n* = 3098). The Kruskal–Wallis test indicated a significant difference in the annual number of conjunctival specimens between the various tenures, *χ*^2^(3) = 64.24, *p* < .001. The effect size (*η*^2^) was 0.91, indicating a large effect. Dunn’s post-hoc test indicated that the mean ranks of the following pairs are significantly different: gr1-gr2 (*p* < .001), gr1-gr3 (*p* < .001), gr1-gr4 (*p* < .001), gr2-gr3 (*p* = .018), gr2-gr4 (*p* < .001).

Alongside the annual numbers of eyelid samples, their average proportion per interval has also increased considerably, quadrupling from 13% in the years 1945–1954 to 54% in 2005–2015 (Fig. 7). Their best fit across our study period was obtained with a second-degree polynomial function (y = − 0.0122x^2^ + 0.1675x—0.0328, *r*^2^ = 0.9948). Within each of the 7 time intervals, the Shapiro–Wilk test did not show a significant departure from normality, in interval 1: *W*(10) = 0.94, *p* = .579, interval 2: *W*(10) = 0.97, *p* = .970, interval 3: *W*(10) = 0.96, *p* = .836, interval 4: *W*(10) = 0.89, *p* = .173, interval 5: *W*(10) = 0.95, *p* = .688, interval 6: *W*(10) = 0.94, *p* = .631, and in interval 7: *W*(11) = 0.95, *p* = .649. Levene’s test for equality of variances was not found to be violated for the present analysis, *F*(6,64) = 1.970, *p* = .083. The effect size (*η*^2^) was 0.16, indicating a large effect. Tukey’s HSD test showed that there is no significant difference between the variances of any pair of intervals. One-way ANOVA revealed significant differences in the mean relative frequency of eyelid specimens examined each year across the 7 intervals, *F*(6,64) = 113.80, *p* < .001. The effect size (*η*^2^) was 0.91, indicating a large effect. Tukey’s HSD test indicated that the means of only the following interval (int) pairs are not significantly different: int4-int5 (*p* = .085), int5-int6 (*p* = .949), int5-int7 (*p* = .703), int6-int7 (*p* = .998), while significant differences were found between the means of any other pairs (*p* < .001).

Within the same time span, the mean interval-based proportion of enucleated eyeballs dropped 38-fold from 61% to just 1.6% (Fig. 7). A second-degree polynomial function (y = 0.0159x^2^—0.2248x + 0.8226, *r*^2^ = 0.9746) provided the best fit for this remarkable decrease across our study period. The Shapiro–Wilk test did not show a significant departure from normality in interval 1: *W*(10) = 0.95, *p* = .768, interval 2: *W*(10) = 0.93, *p* = .511, interval 4: *W*(10) = 0.95, *p* = .712, interval 5: *W*(10) = 0.94, *p* = .622, interval 6: *W*(10) = 0.89, *p* = .154, and in interval 7: *W*(11) = 0.94, *p* = .483. Only in interval 3 the data are not normally distributed: *W*(10) = 0.81, *p* = .022. Levene’s test for equality of variances was found to be violated for the present analysis, *F*(6,64) = 4.735, *p* < .001. The effect size (*η*^2^) was 0.31, indicating a large effect. Tukey’s HSD test showed that the variances of the following pairs of intervals (int) are significantly different: int1-int5 (*p* = .016), int1-int6 (*p* = .006), int1-int7 (*p* = .002), int3-int7 (*p* = .037). The Kruskal–Wallis test revealed a significant difference in the mean relative frequency of enucleated eyeballs between the different intervals, *χ*^2^(6) = 66.67, *p* < .001. The effect size (*η*^2^) was 0.95, indicating a large effect. Dunn’s post-hoc test indicated that the mean ranks of the following pairs are not significantly different: int1-int2 (*p* = .380), int2-int3 (*p* = .135), int2-int4 (*p* = .063), int3-int4 (*p* = .721), int3-int5 (*p* = .067), int4-int5 (*p* = .141), int5-int6 (*p* = .279), int6-int7 (*p* = .244), while significant differences were found between the mean ranks of any other pairs (*p* < .001).

The mean fraction of corneal excisions climbed fivefold from 4.4% to 22% across the intervals of our study period (Fig. 7). A second-degree polynomial fit (y = 0.0008x^2^ + 0.0174x + 0.0303, *r*^2^ = 0.8219) and a linear fit (y = 0.024x + 0.0203, *r*^2^ = 0.8190) provided similar coefficients of determination. The Shapiro–Wilk test indicated no significant departure from normality in interval 1: *W*(10) = 0.87, *p* = .105, interval 2: *W*(10) = 0.93, *p* = .460, interval 3: *W*(10) = 0.96, *p* = .862, interval 4: *W*(10) = 0.96, *p* = .790, interval 5: *W*(10) = 0.97, *p* = .946, and in interval 7: *W*(11) = 0.97, *p* = .959. Only in interval 6 the data are not normally distributed: *W*(10) = 0.77, *p* = .009. Levene’s test for equality of variances was found to be violated for the present analysis, *F*(6,64) = 2.773, *p* = .018. The effect size (*η*^2^) was 0.21, indicating a large effect. Tukey’s HSD test showed that the variances of the following pairs of intervals (int) are significantly different: int2-int3 (*p* = .048), int3-int4 (*p* = .009). The Kruskal–Wallis test revealed a significant difference in the interval-based mean relative frequency of corneal specimens across our study period, *χ*^2^(6) = 46.94, *p* < .001. The effect size (*η*^2^) was 0.64, indicating a large effect. Dunn’s post-hoc test indicated that the mean ranks of the following pairs are not significantly different: int1-int2 (*p* = .927), int2-int3 (*p* = .056), int3-int4 (*p* < .439), int3-int5 (*p* = .167), int3-int6 (*p* < .465), int4-int5 (*p* = .544), int4-int6 (*p* = .965), int5-int6 (*p* = .516), while significant differences were found between the mean ranks of any other pairs (*p* < .001).

The mean fraction of conjunctival specimens in each interval doubled from 7.8% to 16% within our study period (Fig. 7). Coefficients of determination are similar for a second-degree polynomial fit (y = 0.0012x^2^ + 0.004x + 0.0793, *r*^2^ = 0.5769) and for a linear fit (y = 0.014x + 0.0643, *r*^2^ = 0.5634). One-way ANOVA revealed significant differences in the mean relative frequency of conjunctival specimens across the 7 intervals, *F*(6,64) = 16.81, *p* < .001. Tukey’s HSD test indicated that the means of the following interval (int) pairs are not significantly different: int1-int2 (*p* = .972), int1-int4 (*p* = .967), int1-int5 (*p* = .680), int2-int4 (*p* = .999), while a significant difference was found between the means of any other pairs (*p* < .001).

The first temporal artery was biopsied within the 1955–1964 interval, namely in 1959, the proportion of temporal artery excisions plateauing at 8% from the mid-1970s to the mid-1990s, then falling back to just 2.2% in 2005–2015. A third-degree polynomial function (y = − 0.0015x^3^ + 0.0116x^2^—0.0041x—0.0128, *r*^2^ = 0.7544) provided the best fit across our study period. The Kruskal–Wallis test indicated that there is a significant difference in the mean relative frequency of temporal artery biopsies across intervals 3–7 (1965–1974 to 2005–2015), *χ*^2^(4) = 35.64, *p* < .001. Dunn’s post-hoc test indicated that the mean ranks of the following pairs are not significantly different: int3-int6 (*p* = .822), int3-int7 (*p* = .421), int4-int5 (*p* < .928), int6-int7 (*p* = .293), while significant differences were found between the mean ranks of any other pairs (*p* < .001).

The fraction of the combined group “other location” dropped from 14% each during the first two decades to just 4.4% in 2005–2015. A second-degree polynomial fit (y = 0.0007x^2^—0.0215x + 0.1675, *r*^2^ = 0.8558) and a linear fit (y = 0.0161x + 0.1593, *r*^2^ = 0.8513) provided similar coefficients of determination. Statistical evaluations were not performed for this group.

Figure 8 provides an overview on the relative frequency of specimens from the main topographies histologically examined between 1945 and 2015 (Table 3): eyelid (50%), cornea (16%), conjunctiva (14%), eyeball (9.1%), temporal artery (3.8%) and the heterogeneous group “other location” (6.7%). The % values for the individual components of “other location” in this pie-of-pie chart refer to the sum of all topographical areas. It comprises 16 less frequent topographical regions and consists of: orbit (1.4%), lens (0.94%), lacrimal duct (0.78%), iris (0.53%), intraocular tissue (0.47%), vitreous (0.45%), non-ophthalmological locations (0.44%, e.g. nose, forehead etc.), lacrimal gland (0.37%), retina (0.26%), exenteration (0.16%), evisceration (0.15%), sclera (0.10%), anterior chamber angle (0.09%), choroid (0.07%), anterior chamber (0.05%) and optic nerve (0.04%). “Unclear localisation” forms a subgroup that contributes 0.42% and includes samples with unclear origin and/or missing information. In specimens submitted from external medical institutions and practising ophthalmologists between 1994 and 2015, the most frequent location was again the eyelid (27%), followed by cornea (27%), conjunctiva (22%), eyeball (9.2%), temporal artery (0.9%) and the group “other location” (4.2%), while in 9.8% of the samples the location was unclear (not shown).

**Fig. 9:**
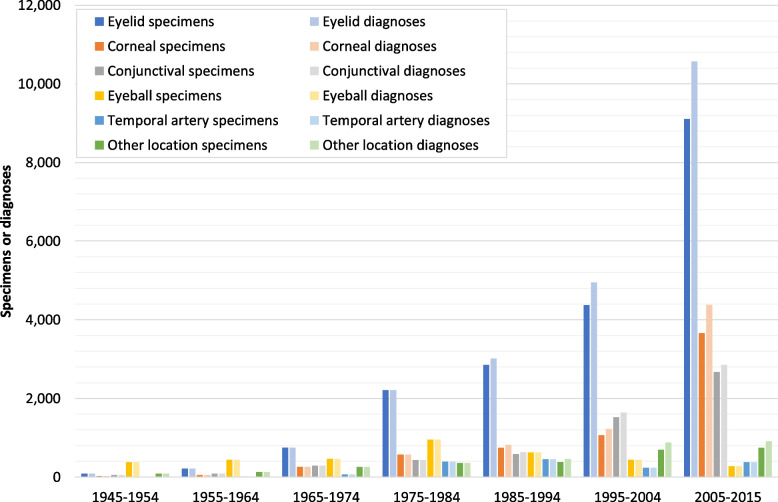
Changes in the number of samples and leading diagnostic categories from main topographical regions. Number of surgical specimens and associated leading histological diagnostic categories from main topographical areas within the 7 intervals of the study period (1945–2015). Other location: see Fig. 8

Changes in the number of samples with associated leading histological diagnostic categories from main topographical regions within the chosen intervals during the study period (1945–2015) are depicted in Fig. 9. Numbers and relative frequencies of the corresponding leading histological diagnostic categories in each time interval between 1945 and 2015 are shown in Table 4. From 1945 to 1984, one diagnostic category was assigned to each sample. In the 1985–1994 period, 361 additional diagnostic categories (6.4%) were associated with eyelids (+ 162), cornea (+ 79), conjunctiva (+ 46) and with other locations (+ 74). In 1995–2004, the sum of assigned diagnostic categories outnumbered the sum of specimens by 1022 (12%), which were again distributed across the eyelids (+ 568), cornea (+ 155), conjunctiva (+ 118) and other sites (+ 181). Finally, from 2005 to 2015, 19,358 diagnostic categories exceeded 16,828 total case numbers by 2,530 (15%), attributable to eyelids (+ 1,461), cornea (+ 726), conjunctiva (+ 174) and other topographical regions (+ 169). Throughout the study period, eyeball and temporal artery were assigned only to one leading histological diagnostic category.

**Table 4:** Changes in leading diagnostic categories in main topographical regions

Time period	1945–1954	1955–1964	1965–1974	1975–1984	1985–1994	1995–2004	2005–2015	Total number
Eyelid	83 (13%)	210 (23%)	746 (36%)	2,206 (45%)	3,009 (50%)	4,942 (53%)	10,568 (55%)	21,764 (50%)
Cornea	27 (4.4%)	49 (5.4%)	260 (13%)	568 (12%)	817 (14%)	1,216 (13%)	4,382 (23%)	7,319 (17%)
Conjunctiva	48 (7.8%)	83 (9.2%)	287 (14%)	431 (8.8%)	628 (10%)	1,637 (18%)	2,849 (15%)	5,963 (14%)
Eyeball	374 (61%)	438 (48%)	462 (22%)	945 (19%)	624 (10%)	438 (4.7%)	274 (1.4%)	3,555 (8.2%)
Temporal artery	0 (0%)	1 (0.1%)	64 (3.1%)	389 (7.9%)	451 (7.5%)	235 (2.5%)	377 (1.9%)	1,517(3.5%)
Other location	86 (14%)	125 (14%)	251 (12%)	357 (7.3%)	454 (7.6%)	870 (9.3%)	908 (4.7%)	3,051 (7.1%)
Sum	618	906	2,070	4,896	5,983	9,338	19,358	43,169

Number and relative frequency of leading histological diagnostic categories in main topographical areas within 10-year intervals (2005–2015: 11 years) during the observation period (1945–2015). Other location: orbit, lens, lacrimal duct, iris, vitreous, lacrimal gland, retina, exenteration, choroid, evisceration, sclera, optic nerve and further sites, see chapter “Results” and Fig. 8. Percentages may not total 100 due to rounding

#### Eyelid

Our database contains 19,573 eyelid specimens, based on which 21,764 diagnoses were made. Starting with just 1 specimen (2.1%) of 47 total surgical samples in 1945, the number of eyelid lesions increased steadily to 445 (50%) of 889 overall in 2002, then to 888 (59%) of 1505 total lesions in 2010, finally reaching 1043 (51%) eyelid lesions of 2046 total lid diagnoses in 2015 (not explicitly shown; see also Fig. 2).

Within the study period, the most frequent diagnostic category were benign tumours (*n* = 8131; 37%), followed by inflammations (*n* = 5,025; 23%), malignant tumours (*n* = 4396; 20%), and “other eyelid diagnosis” (*n* = 4212; 19%) (Table 5). The number of benign tumours increased 95-fold from 34 (41%) of 83 eyelid lesions in 1945–1954 to 3,241 (31%) of 10,568 lesions in 2005–2015, clearly outnumbering malignant tumours counting 35 (42%) in 1945–1954 and, after a steady increase, 1969 (19%) of all eyelid lesions in 2005–2015. Inflammations increased 60-fold within the first four decades from 12 (14%) of 83 diagnoses in 1945–1954 to 723 (24%) of 3009 diagnoses in 1985–1994, then to 2,606 (25%) of 10,568 lesions in total in 2005–2015. “Other eyelid diagnosis” climbed from initially only 2 cases (2.4%) in 1945–1954 to 280 (9.3%) in 1985–1994, finally amounting to notable 2752 diagnoses (26%) of all eyelid lesions in 2005–2015.

**Table 5:** Changes in eyelid diagnostic categories

Time period	1945–1954	1955–1964	1965–1974	1975–1984	1985–1994	1995–2004	2005–2015	Total number
Benign tumour	34 (41%)	91 (44%)	395 (53%)	1,277 (58%)	1,368 (45%)	1,725 (35%)	3,241 (31%)	8,131 (37%)
Inflammation	12 (14%)	30 (14%)	112 (15%)	335 (15%)	723 (24%)	1,207 (24%)	2,606 (25%)	5,025 (23%)
Malignant tumour	35 (42%)	77 (37%)	215 (29%)	512 (23%)	638 (21%)	950 (19%)	1,969 (19%)	4,396 (20%)
Other eyelid diagnosis	2 (2.4%)	12 (5.7%)	24 (3.2%)	82 (3.7%)	280 (9.3%)	1,060 (21%)	2,752 (26%)	4,212 (19%)
Sum	83	210	746	2,206	3,009	4,942	10,568	21,764

Number and relative frequency of eyelid lesions with associated diagnostic categories within 10-year intervals (2005–2015: 11 years) during the observation period (1945–2015). Other eyelid diagnoses: see Table 7. Percentages may not total 100 due to rounding

A total of 8,131 benign eyelid tumour diagnoses were classified according to their cellular origin, the most common benign tumour being of epithelial origin (*n* = 5936; 73%), followed by melanocytic (*n* = 1201; 15%), inflammatory (*n* = 403; 5%) and mesenchymal tumours (*n* = 347; 4.3%), and finally choristoma, neuronal, lymphocytic and “other benign lid tumours” (sum: *n* = 244; 3%) (Table 6). 4396 malignant lid tumours were classified based on existing metastatic potential (EMP) and absent metastatic potential (AMP). The most common of 472 EMP-tumours is squamous cell carcinoma (*n* = 265; 56%), followed by sebaceous gland carcinoma (*n* = 75; 16%), lymphoma (*n* = 50; 11%), melanoma (*n* = 36; 7.6%), other lid carcinoma (*n* = 18; 3.8%), metastasis (*n* = 15; 3.2%), sarcoma, adenocarcinoma and malignant transformation (sum: *n* = 13; 2.6%). Basal cell carcinomas were categorised as AMP-tumours since the periocular lesion in almost all cases does not reach the size of lesions with metastatic potential [38]. Basal cell carcinoma (*n* = 3768; 96%) was the most frequent among 3924 AMP-tumours, followed by squamous epithelial precancerous lesions (*n* = 114; 2.9%), while only 42 specimens (1.1%) with melanocytic precancerous lesion were found.

**Table 6:** Benign and malignant tumours of the eyelid

Origin/category of benign tumour	n	(%)	Malignant EMP-tumour entity	n	(%)	Malignant AMP-tumour entity	n	(%)
Epithelial	5,936	73	Squamous cell carcinoma	265	56	Basal cell carcinoma	3,768	96
Melanocytic	1,201	15	Sebaceous gland carcinoma	75	16	Squamous epithelial precancerous lesion	114	2.9
Inflammatory	403	5.0	Lymphoma	50	11	Melanocytic precancerous lesion	42	1.1
Mesenchymal	347	4.3	Melanoma	36	7.6			
Choristoma	140	1.7	Other lid carcinoma	18	3.8			
Other benign lid tumour	62	0.8	Metastasis	15	3.2			
Neuronal	33	0.4	Sarcoma	11	2.2			
Lymphocytic	9	0.1	Malignant transformation	2	0.4			
Sum	8,131		Sum	472		Sum	3,924	

Number and relative frequency of eyelid lesions with associated benign and malignant tumour diagnoses during the observation period (1945–2015). Malignant tumours were classified based on existing metastatic potential (EMP), absent metastatic potential (AMP) and on their cell of origin. Percentages may not total 100 due to rounding

We finally formed a heterogeneous group “other eyelid diagnosis” comprising 4212 samples from eight diagnostic categories (Table 7), which includes 1784 (42%) normal findings, 1533 (36%) tumour resection specimens, 546 (13%) degenerative changes, 249 (5.9%) scar tissues, and further 57 tumours of unknown origin, 33 traumata, 7 unclear diagnoses and 3 cases of infection (sum: 2.5%). Subcategory “tumour resection specimen” includes specimens obtained in secondary surgery after R1 resection.

**Table 7:** Other eyelid diagnosis

Diagnostic category	n	(%)
Normal finding	1784	42
Tumour resection specimen	1533	36
Degeneration	546	13
Scar tissue	249	5.9
Tumour of unknown origin	57	1.4
Trauma	33	0.8
Unknown diagnosis	7	0.2
Infection	3	0.1
Sum	4212	

Number and relative frequency of eyelid lesions classified as “other eyelid diagnosis” during the observation period (1945–2015). Percentages may not total 100 due to rounding

#### Cornea

Six thousand, three hundred fifty-nine corneal samples with 7319 corresponding histological diagnoses are registered in our database. Across the entire study period, the number of corneal lesions rose from 7 cases (15%) of 47 in total in 1945 continuously to 104 (12%) of 889 overall in 2002, then increased fivefold to 536 (26%) of 2046 total lesions in 2015 (not explicitly shown; see also Fig. 2). This last, quite pronounced increase can primarily be attributed to an almost sixfold increase in the number of dystrophies, a ninefold rise in failed transplant specimens and a fourfold increase in scar tissue samples (Table 8). During the observation period, the most frequent diagnostic categories were corneal dystrophy (*n* = 1580; 22%), keratoconus (*n* = 975; 13%), inflammation (*n* = 832; 11%), transplant failure (*n* = 806; 11%), scarring (*n* = 730; 10%), bullous keratopathy (*n* = 713; 9.7%) and “other corneal diagnosis” (*n* = 1242; 17%). Between 1945 and 1954, only 27 corneal samples were sent to our ophthalmic pathology laboratory which were primarily ulcers (*n* = 7; 26%), inflammations (*n* = 6; 22%) and malignant tumours (*n* = 5; 19%). As their numbers increased over time, their proportion noticeably declined to just 5.6% (*n* = 246), 10% (*n* = 435) and 0.1% (*n* = 6) respectively between 2005 and 2015.

**Table 8:** Changes in corneal diagnostic categories

Time period	1945–1954	1955–1964	1965–1974	1975–1984	1985–1994	1995–2004	2005–2015	Total number
Dystrophy	0 (0%)	0 (0%)	38 (15%)	78 (14%)	92 (11%)	201 (17%)	1,171 (27%)	1,580 (22%)
Keratoconus	0 (0%)	2 (4.1%)	33 (13%)	122 (21%)	155 (19%)	217 (18%)	446 (10%)	975 (13%)
Inflammation	6 (22%)	13 (27%)	29 (11%)	107 (19%)	77 (9.4%)	165 (14%)	435 (10%)	832 (11%)
Transplant failure	4 (15%)	3 (6.1%)	28 (11%)	55 (10%)	48 (5.9%)	65 (5.3%)	603 (14%)	806 (11%)
Scar tissue	2 (7.0%)	7 (14%)	76 (29%)	89 (16%)	101 (12%)	87 (7.1%)	368 (8.4%)	730 (10%)
BK w/o cataract surgery	0 (0%)	0 (0%)	0 (0%)	9 (1.6%)	23 (2.8%)	128 (11%)	288 (6.6%)	448 (6.1%)
BK w/cataract surgery	0 (0%)	0 (0%)	4 (1.5%)	29 (5.1%)	114 (14%)	39 (3.2%)	79 (1.8%)	265 (3.6%)
Ulcer	7 (26%)	4 (8.2%)	7 (2.7%)	19 (3.4%)	35 (4.3%)	93 (7.6%)	246 (5.6%)	411 (5.6%)
Malignant tumour	5 (19%)	3 (6.1%)	2 (0.8%)	1 (0.2%)	5 (0.6%)	8 (0.7%)	6 (0.1%)	30 (0.4%)
Other corneal diagnosis	3 (11%)	17 (35%)	43 (17%)	59 (10%)	167 (20%)	213 (18%)	740 (17%)	1,242 (17%)
Sum	27	49	260	568	817	1,216	4,382	7,319

Number and relative frequency of corneal lesions with associated diagnoses within 10-year intervals (2005–2015: 11 years) during the observation period (1945–2015). Other corneal diagnosis: see Table 11. Percentages may not total 100 due to rounding. BK = bullous keratopathy; with and without previous cataract surgery

A total of 1,580 corneal dystrophies were diagnosed in our laboratory between 1965 and 2015, while no such cases were reported before. Submissions steadily increased from 38 (15%) of 260 corneal lesions in 1965–1974 to 201 (17%) of 1216 diagnoses in 1995–2004, followed by the aforementioned sixfold rise to 1,171 dystrophies (27% of *n* = 4382) in 2005–2015 (Table 8), with Fuchs’ endothelial dystrophy being the most common, as expected. In decade 1965–1974, Fuchs’ dystrophy already accounted for 55% (*n* = 21) of all corneal dystrophies (Table 9); its proportion grew to 92% (*n* = 1082) within the last observation interval. The Kruskal–Wallis test indicated that there is a significant difference in the mean relative frequency of Fuchs’ dystrophies among the corneal lesions across intervals 3–7 (1965–1974 to 2005–2015), *χ*^2^(4) = 25.26, *p* < .001. Dunn’s post-hoc test indicated that the mean ranks of the following pairs are significantly different: int3-int7 (*p* < .001), int4-int7 (*p* < .001), int5-int7 (*p* < .001), int6-int7 (*p* < .021).

**Table 9:** Changes in corneal dystrophies

Time period	1945–1954	1955–1964	1965–1974	1975–1984	1985–1994	1995–2004	2005–2015	Total number
Fuchs’ dystrophy	0 (0%)	0 (0%)	21 (55%)	50 (71%)	62 (69%)	150 (75%)	1,082 (92%)	1,365 (86%)
Macular dystrophy	0 (0%)	0 (0%)	3 (7.9%)	9 (13%)	12 (13%)	7 (3.5%)	17 (1.4%)	48 (3.0%)
Lattice dystrophy	0 (0%)	0 (0%)	5 (13%)	6 (8.6%)	0 (0%)	11 (5.5%)	20 (1.7%)	42 (2.7%)
Granular dystrophy	0 (0%)	0 (0%)	4 (11%)	2 (2.9%)	6 (6.7%)	15 (7.5%)	14 (1.2%)	41 (2.6%)
Other corneal dystrophy	0 (0%)	0 (0%)	5 (13%)	3 (4.3%)	10 (11%)	18 (9.0%)	48 (4.1%)	84 (5.3%)
Sum	0	0	38	70	90	201	1,181	1,580

Number and relative frequency of various corneal dystrophies within 10-year intervals (2005–2025: 11 years) during the observation period (1945–2015). Other corneal dystrophy: see text. Percentages may not total 100 due to rounding

Macular, lattice, granular and other corneal dystrophies accounted for the remaining 45% (*n* = 17) of 38 diagnoses in 1965–1974, dropping to just 8.4% (*n* = 99) of 1,181 diagnoses in 2005–2015. Subgroup “other corneal dystrophy” includes Avellino dystrophy, Franceschetti dystrophy, gelatinous dystrophy, Gly623 Asp TGFBi-mutation associated dystrophy, Map-Dot-Fingerprint dystrophy, Meesmann dystrophy, Lisch dystrophy, posterior polymorphous dystrophy, Reis-Bücklers dystrophy, Schnyder dystrophy, subepithelial mucinous dystrophy, and Thiel-Behnke dystrophy.

With a total 975 histological findings of keratoconus, the first 2 diagnoses (4.1%) of 49 corneal lesions were reported between 1955 and 1964, followed by a steady increase to 446 (10%) of 4,382 corneal cases in 2005–2015 (Table 8). From the fourth decade until 2015, however, their percentage declined. As expected, we see predominantly male patients in each of the 10-year intervals since 1955 (Table 10), steadily increasing over time and amounting to 331 (74%) of 446 keratoconus cases in 2005–2015. With 288 (30%) female vs 676 (69%) male patients, the relative frequency of keratoconus in women was 2.3 times lower overall. Starting with 8 diagnoses in females in 1965–1974, their number reached 110 (25%) of 446 cases in 2005–2015. In 11 keratoconus cases (1.1%) across the study period, the patients’ sex was not documented in our archive.

**Table 10:** Changes in keratoconus by gender

Time period	1945–1954	1955–1964	1965–1974	1975–1984	1985–1994	1995–2004	2005–2015	Total number
Female patients	0 (0%)	0 (0%)	8 (24%)	47 (39%)	48 (31%)	75 (35%)	110 (25%)	288 (30%)
Male patients	0 (0%)	2 (100%)	25 (76%)	71 (58%)	107 (69%)	140 (65%)	331 (74%)	676 (69%)
Gender not documented	0 (0%)	0 (0%)	0 (0%)	4 (3.0%)	0 (0%)	2 (0.9%)	5 (1.1%)	11 (1.1%)
Sum	0	2	33	122	155	217	446	975

Number and relative frequency of keratoconus specimens from female and male patients received within 10-year intervals (2005–2025: 11 years) during the observation period (1945–2015). Percentages may not total 100 due to rounding

Inflammations steadily increased more than 70-fold in number from 6 (22%) of 27 corneal lesions within the first decade to 435 (10%) of 4,382 lesions in 2005–2015 (Table 8). Following a rather gentle increase across the first six decades, the number of failed transplants rose sharply after 2004, increasing almost tenfold to 603 cases (14%). This 2005–2015 interval thus accounted for 75% of 806 transplant failures in total, representing 11% of all corneal lesions recorded within the study period.

Bullous keratopathies (BK) were divided into two individual groups, those with previous cataract surgery (BK w/cataract surgery; *n* = 265; 3.6%) and those without (BK w/o cataract surgery; *n* = 448; 6.1%); BK do not appear in our archive before 1965 (Table 8). Until the fifth decade, bullous keratopathies after cataract surgery clearly predominated, but then their ratio reversed in favour of such BK without previous cataract surgery taking a more than 12-fold increase from 23 (2.8%) in 1985–1994 to 288 diagnoses (6.6%) in 2005–2015.

Only 2 (7.0%) of 27 corneal samples submitted in the first decade were scar tissue, while already 76 (29%) of 261 corneal lesions submitted 1965–1974 were diagnosed with scarring (Table 8). Scar tissue took a fourfold increase from 87 diagnoses (7.1%) in 1995–2004 to 368 scar lesions (8.4% of *n* = 4,382) in the 2005–2015 interval, amounting to 730 diagnoses (10%) of *n* = 7,319 corneal lesions during the 71-year time span. The number of corneal ulcers climbed from 7 (26%) in 1945–1954 to 246 (5.6%) across our study period, totalling *n* = 411 diagnoses (Table 8).

A heterogeneous group “other corneal diagnosis” summarises 1,242 corneal lesions (17%) from eight diagnostic categories: degeneration (*n* = 704; 57%), infection (*n* = 253; 20%), trauma (*n* = 115; 9.3%), normal finding (*n* = 96; 7.7%), benign tumour (*n* = 65; 5.2%), unclear diagnosis (*n* = 5; 0.4%), tumour of unknown origin (*n* = 3; 0.2%) and congenital malformation (*n* = 1; 0.1%) (Table 11). The most common among 65 benign corneal tumours was choristoma (*n* = 37; 57%), followed by epithelial (*n* = 20; 31%), mesenchymal (*n* = 4; 6.2%) and melanocytic tumours (*n* = 4; 6.2%) (Table 12). 30 malignant tumours were classified based on existing and absent metastatic potential into EMP- and AMP-tumours. Squamous cell carcinoma (*n* = 8; 73%) is the most prevalent of 11 EMP-tumours, followed by melanoma (*n* = 3; 27%). Among 19 AMP-tumours, the squamous epithelial precancerous lesion was the most frequent (*n* = 18; 95%), followed by 1 corneal lesion (5.3%) representing a basal cell carcinoma.

**Table 11:** Other corneal diagnosis

Diagnostic category	n	(%)
Degeneration	704	57
Infection	253	20
Trauma	115	9.3
Normal finding	96	7.7
Benign tumour	65	5.2
Unclear diagnosis	5	0.4
Tumour of unknown origin	3	0.2
Congenital malformation	1	0.1
Sum	1,242	

Number and relative frequency of corneal lesions classified as “other corneal diagnosis” during the observation period (1945–2015). Percentages may not total 100 due to rounding

**Table 12:** Benign and malignant tumours of the cornea

Origin/category of benign tumour	n	(%)	Malignant EMP-tumour entity	n	(%)	Malignant AMP-tumour entity	n	(%)
Choristoma	37	57	Squamous cell carcinoma	8	73	Squamous epithelial precancerous lesion	18	95
Epithelial	20	31	Melanoma	3	27	Basal cell carcinoma	1	5.3
Mesenchymal	4	6.2						
Melanocytic	4	6.2						
Sum	65		Sum	11		Sum	19	

Number and relative frequency of corneal lesions with associated benign and malignant tumour diagnoses during the observation period (1945–2015). Malignant tumours were classified based on existing metastatic potential (EMP), absent metastatic potential (AMP) and on their cell of origin. Percentages may not total 100 due to rounding

#### Conjunctiva

Five thousand six hundred twenty-five conjunctival samples with a total of 5,963 diagnoses are archived at our ophthalmic pathology lab. There was no case in 1945; the first 4 conjunctival lesions (5.4%) of 74 cases in total were submitted in 1946. Their number very gradually increased at an average rate of 1.1 cases per year to 24 lesions (22%) of 107 in total in 1967 and to 64 (12%) of 556 overall in 1979. After some decline to 25 cases (6.2% of *n* = 405) in 1987, we found a 12-fold increase to 309 (15%) of 2,046 total lesions in 2015 (not explicitly shown; see also Fig. 2). The most frequent diagnostic categories within the study period were degeneration (*n* = 2,159; 36%), benign tumour (*n* = 1,578; 26%) and inflammation (*n* = 966; 16%), followed by malignant tumour (*n* = 666; 11%) and “other conjunctival diagnosis” (*n* = 594; 10%) (Table 13).

**Table 13:** Changes in conjunctival diagnostic categories

Time period	1945–1954	1955–1964	1965–1974	1975–1984	1985–1994	1995–2004	2005–2015	Total number
Degeneration	3 (6.3%)	6 (7.2%)	51 (18%)	58 (13%)	173 (28%)	597 (37%)	1,271 (45%)	2,159 (36%)
Benign Tumour	18 (38%)	25 (30%)	139 (48%)	218 (51%)	213 (34%)	356 (22%)	609 (21%)	1,578 (26%)
Inflammation	21 (44%)	35 (42%)	60 (21%)	70 (16%)	119 (19%)	294 (18%)	367 (13%)	966 (16%)
Malignant Tumour	6 (13%)	16 (19%)	26 (9.1%)	57 (13%)	44 (7.0%)	155 (9.5%)	362 (13%)	666 (11%)
Other conjunctival diagnosis	-	1 (1.2%)	11 (3.8%)	28 (6.5%)	79 (13%)	235 (14%)	240 (8.4%)	594 (10%)
Sum	48	83	287	431	628	1,637	2,849	5,963

Number and relative frequency of conjunctival lesions with associated diagnoses received within 10-year intervals (2005–2015: 11 years) during the observation period (1945–2015). Other conjunctival diagnosis: see Table 15. Percentages may not total 100 due to rounding

While the absolute number in each diagnostic category increased steadily from 1945 to 2015, their relative proportions changed quite pronouncedly (Table 13). Within our study period, the relative frequency of a total of *n* = 2,159 conjunctival degenerative changes notably increases from 6.3% (*n* = 3) in 1945–1954 to 45% (*n* = 1,271) in 2005–2015. We see conjunctival inflammations (*n* = 966) and benign tumours (*n* = 1,578) initially predominate in 1945–1954, accounting for 44% (*n* = 21) and 38% (*n* = 18) respectively of 48 conjunctival lesions. Then they decline in proportion to 13% (*n* = 367) and 21% (*n* = 609), respectively, of 2,849 conjunctival diagnoses in 2005–2015. The proportion of malignant tumours is 13% (*n* = 6) in 1945–1954, 13% (*n* = 57) in 1975–1984 and again 13% in 2005–2015, however now based on 362 lesions, totalling *n* = 666 in the entire period observed. The 594 “other conjunctival diagnoses” climbed from 1.2% (*n* = 1) in 1955–1964 to 14% (*n* = 235) in 1995–2004, finally dropping to 8.4% (*n* = 240) in the last interval (2005–2015).

The most common of 1,578 benign conjunctival tumours are of melanocytic origin (*n* = 772; 49%), followed by epithelial (*n* = 653; 41%) and mesenchymal tumours (*n* = 70; 4.4%), choristoma (*n* = 60; 3.8%), lymphocytic tumours (*n* = 18; 1.1%) and other benign conjunctival tumours (*n* = 5; 0.3%) (Table 14). Among 313 malignant EMP-tumours, melanoma is the most prevalent (*n* = 142; 45%), followed by lymphoma (*n* = 79; 25%), squamous cell carcinoma (*n* = 78; 25%) and other malignant conjunctival tumours (*n* = 14; 4.5%). Within 353 malignant AMP-tumours, the most frequent are epithelial precancerous lesions (*n* = 305; 86%), followed by melanocytic precancerous lesions (*n* = 45; 13%) and basal cell carcinomas (*n* = 3; 0.8%).

**Table 14:** Benign and malignant tumours of the conjunctiva

Origin/category of benign tumour	n	(%)	Malignant EMP-tumour entity	n	(%)	Malignant AMP-tumour entity	n	(%)
Melanocytic	772	49	Melanoma	142	45	Epithelial precancerous lesion	305	86
Epithelial	653	41	Lymphoma	79	25	Melanocytic precancerous lesion	45	13
Mesenchymal	70	4.4	Squamous cell carcinoma	78	25	Basal cell carcinoma	3	0.8
Choristoma	60	3.8	Other malignant conjunctival tumour	14	4.5			
Lymphocytic	18	1.1						
Other benign conjunctival tumour	5	0.3						
Sum	1,578		Sum	313		Sum	353	

Number and relative frequency of conjunctival lesions with associated benign and malignant tumour diagnoses during the observation period (1945–2015). Malignant tumours were classified based on existing metastatic potential (EMP), absent metastatic potential (AMP) and on their cell of origin. Percentages may not total 100 due to rounding

A heterogeneous group of “other conjunctival diagnosis” combines seven subcategories and includes 594 (10%) of all conjunctival diagnoses: normal findings (*n* = 210; 35%), scar tissues without previous surgery (*n* = 164; 28%), various postoperative sequelae (*n* = 150; 25%), tumours of unknown origin (*n* = 30; 5.1%), traumata (*n* = 28; 4.7%) unclear diagnoses (*n* = 9; 1.5%) and infections (*n* = 3; 0.5%) (Table 15).

**Table 15:** Other conjunctival diagnosis

Diagnostic category	n	(%)
Normal finding^a^	210	35
Scar tissue without previous surgery	164	28
Various postoperative sequelae^b^	150	25
Tumour of unknown origin	30	5.1
Trauma	28	4.7
Diagnosis unclear	9	1.5
Infection	3	0.5
Sum	594	

Number and relative frequency of conjunctival lesions classified as “other conjunctival diagnosis” during the observation period (1945–2015). Percentages may not total 100 due to rounding

^a^e.g. normal conjunctiva after resection following tumour removal, control specimens removed for studies

^b^e.g. filtering bleb, scar tissue

#### Eyeball

Three thousand, five hundred fifty-five eyeball specimens from our archive were examined and 3,555 leading histological diagnostic categories or causes for enucleation were established. The relative frequency of surgically enucleated eyeballs decreased remarkably from 77% in 1945 to 1.2% in 2015. Starting with 36 enucleations out of a total of 47 specimens in 1945, their annual number remained relatively consistent throughout the study period (median: 43 specimens p.a.; range: 14–121 specimens p.a.), with more frequent occurrences between 1973 and 1994 (median: 77 specimens p.a.; range: 34–121 specimens p.a.). The highest annual number was 121 enucleations (22%) out of a total of 546 lesions in 1981. Consecutively, the figures fell to 43 enucleations (4.8%) of 889 total cases in 2002 and to just 24 (1.2%) of *n* = 2,046 total lesions in 2015 (not explicitly shown; see also Fig. 2). The most frequent leading diagnostic categories as underlying cause for enucleation were trauma (*n* = 871; 25%), followed by malignant tumour (*n* = 691; 19%), glaucoma (*n* = 305; 8.6%), vascular disease (*n* = 297; 8.4%), bulbar inflammation (*n* = 237; 6.7%), postoperative complication (*n* = 236; 6.6%), retinal detachment (*n* = 147; 4.1%), phthisis (*n* = 83; 2.3%) and “other eyeball category” (*n* = 688; 19%) (Table 16).

**Table 16:** Changes in eyeball diagnostic categories

Time period	1945–1954	1955–1964	1965–1974	1975–1984	1985–1994	1995–2004	2005–2015	Total number
Trauma	106 (28%)	109 (25%)	110 (24%)	283 (30%)	161 (26%)	46 (10%)	56 (20%)	871 (25%)
Malignant tumour	53 (14%)	96 (22%)	135 (29%)	183 (19%)	110 (18%)	84 (19%)	30 (11%)	691 (19%)
Glaucoma	53 (14%)	48 (11%)	58 (13%)	54 (5.7%)	51 (8.2%)	37 (8.4%)	4 (1.4%)	305 (8.6%)
Vascular disease	6 (1.6%)	8 (1.8%)	14 (3.0%)	101 (11%)	86 (14%)	42 (10%)	40 (14%)	297 (8.4%)
Bulbar inflammation	31 (8.3%)	29 (6.6%)	26 (5.6%)	53 (5.6%)	41 (6.6%)	27 (6.2%)	30 (11%)	237 (6.7%)
Postoperative complication	17 (4.6%)	14 (3.2%)	10 (2.2%)	46 (4.9%)	39 (6.3%)	62 (14%)	48 (17%)	236 (6.6%)
Retinal detachment	3 (0.8%)	10 (2.3%)	20 (4.3%)	30 (3.2%)	34 (5.5%)	35 (8.0%)	15 (5.4%)	147 (4.1%)
Phthisis	11 (2.9%)	8 (1.8%)	5 (1.1%)	8 (0.8%)	5 (0.8%)	38 (8.7%)	8 (2.9%)	83 (2.3%)
Other eyeball category	94 (25%)	116 (27%)	84 (18%)	187 (20%)	97 (15%)	67 (15%)	43 (17%)	688 (19%)
Sum	374	438	462	945	624	438	274	3,555

Number and relative frequency of eyeballs with associated leading histological diagnostic categories within 10-year intervals (2005–2015: 11 years) during the observation period (1945–2015). Other eyeball category: see Table 17. Percentages may not total 100 due to rounding

During the first 4 decades, we notice an increase in traumata, malignant tumours, vascular diseases, bulbar inflammations, retinal detachments and in cases summarised under “other eyeball category”, generally by a factor of two to three. In vascular diseases, we observe an even 17-fold rise, while for retinal detachment the growth is tenfold. A particular increase in numbers is frequently seen between the periods 1965–1974 and 1975–1984. The relative frequency of glaucoma as underlying cause for enucleation decreases from 14% (*n* = 53) in 1945 to 1.4% (*n* = 4) during the 2005–2015 interval. Both the number and the relative frequency of postoperative complications increased threefold during the observation period. The percentage of phthisis remains between 0.8% and 3% throughout our study, with an exception of 38 cases (8.7%) in 1995–2004.

The heterogeneous group of “other eyeball category” summarises eight subcategories and includes 688 (19%) of all leading eyeball diagnoses as underlying cause for enucleation: corneal pathologies (*n* = 207; 30%), complications of diabetes (*n* = 112; 16%), congenital anomalies (*n* = 86; 13%), uveal pathologies (*n* = 79; 11%), iris pathologies (*n* = 79; 11%), retinal pathologies (*n* = 76; 11%), scleritis (*n* = 28; 4.1%) and M. Coats (*n* = 21; 3.1%) (Table 17).

**Table 17:** Other eyeball category

Diagnostic category	n	(%)
Corneal pathologies	207	30
Complications of diabetes	112	16
Congenital anomalies	86	13
Uveal pathologies	79	11
Iris pathologies	79	11
Retinal pathologies	76	11
Scleritis	28	4.1
M. Coats	21	3.1
Sum	688	

Number and relative frequency of other leading histological diagnostic categories of eyeball lesions (enucleations) during the observation period (1945–2015). Percentages may not total 100 due to rounding

#### Temporal artery

Between 1945 and 2015, a total of 1,517 diagnoses were recorded on 1,517 excised specimens of the temporal artery. One first sample (0.9%) of 115 specimens overall was examined in 1959, the next in 1965 (0.9% of *n* = 111) and yet another temporal artery specimen in 1967 (0.9% of *n* = 107) before the case numbers started to gradually increase to 10 samples (4.6%) of 217 total lesions in 1969. 74 diagnoses (14% of *n* = 523) in 1984 represent the maximum annual number. After 1985, the number of cases declined to between 16 and 59 arteries per year, reaching 53 (2.6%) out of 2,046 total cases in 2015 (see also Fig. 2). The most frequent diagnostic categories within the observed period were degenerative changes (*n* = 574; 38%), inflammation (*n* = 426; 28%), and normal finding (*n* = 517; 34%) (Table 18). Of 64 biopsies in 1965–1974, 28 (44%) showed degenerative changes (mainly arteriosclerosis), 14 (22%) an inflammation and 22 (34%) were normal findings. This pattern changed in 1985–1994, where out of 451 temporal artery biopsies, 85 (19%) showed degenerative changes, 254 (56%) inflammation and 112 (25%) were normal findings. In the last interval of the study period, 182 (48%) out of 377 biopsies were degenerative, 27 (7%) showed an inflammation and 168 (45%) were normal findings.

**Table 18:** Changes in temporal artery diagnostic categories

Time period	1945–1954	1955–1964	1965–1974	1975–1984	1985–1994	1995–2004	2005–2015	Total number
Degeneration	0 (0%)	0 (0%)	28 (44%)	218 (56%)	85 (19%)	61 (26%)	182 (48%)	574 (38%)
Inflammation	0 (0%)	1 (100%)	14 (22%)	71 (18%)	254 (56%)	59 (25%)	27(7%)	426 (28%)
Normal finding	0 (0%)	0 (0%)	22 (34%)	100 (26%)	112 (25%)	115 (49%)	168 (45%)	517 (34%)
Sum	0	1	64	389	451	235	377	1,517

Number and relative frequency of temporal artery diagnostic categories within 10-year intervals (2005–2015: 11 years) during the observation period (1945–2015). Percentages may not total 100 due to rounding

## Discussion

### Specimen numbers

Both the temporal proximity to the Second World War and the destruction of the Freiburg Eye Clinic in 1944 [6] accounted for the comparatively low number of samples received for diagnosis at the beginning of the observation period. The limited capacity of the ophthalmic pathology laboratory, due to its provisional re-commissioning in early December 1944 after the eye hospital was evacuated to a sanatorium and the prioritisation of basic patient care, was mainly responsible for the examination of only 42 to 81 specimens annually until 1954. While the staff gradually became accustomed to the unusual circumstances of the clinic’s temporary relocation, numbers gradually started to rise, reaching 75 to 115 specimens each year between 1955 and 1964. This trend intensified since commissioning of the newly constructed Freiburg Eye Center with its new histology laboratory at a different location to the former, destroyed eye hospital under Wilhelm Wegner in 1964. Hanns-Hellmuth Unger, ophthalmologist at the Freiburg Eye Clinic since 1949, whose focus was on ophthalmopathological research in the course of which he habilitated in 1957, deserves particular mention in this context.

After Günter Mackensen assumed management, annual numbers of specimens increased significantly (*p* < .001) from 154 samples in 1968 to 556 specimens submitted in 1979 (Fig. 2). Mackensen, however, was no ophthalmic pathologist. Already from 1953 to 1967, as a senior physician with Heinrich Harms in Tübingen, Germany, he had particularly been interested in clinical science, where he devoted himself to the refinement of surgical techniques. Consequently, yet again beneficial to Freiburg ophthalmic pathology, Mackensen introduced microsurgical techniques [6] enabling biopsies to be taken more frequently from various topographical areas.

But it was Heinrich Witschel who, when joining the ophthalmic pathology laboratory in Freiburg in 1971, brought about another noticeable increase in case numbers. Even while he spent two years (1974–75) at the Armed Forces Institute of Pathology, Washington, D.C., USA, deepening his expertise in ophthalmic pathology with Lorenz Zimmerman, this only briefly led to a modest decline in the number of specimens examined in 1975. As Witschel left Freiburg in 1984 to assume directorship of the University Eye Clinic in Berlin-Steglitz, sample numbers again dropped only moderately from 523 to 405 specimens in 1987. When Witschel finally returned as head of the Freiburg Eye Clinic in 1988, annual numbers took another unabated surge, significantly increasing (*p* = .017) from 502 to 889 samples in 2002, the year of his retirement (Fig. 2). As early as 1992, Witschel was joined by Karin Loeffler as head of the ophthalmic pathology lab, who habilitated in 1995 and has since become a recognised ophthalmic pathologist and long-standing chairwoman of the Association of German-speaking Ophthalmic Pathologists (DOP).

One of the authors, Claudia Auw-Haedrich, succeeded her in 1996, after having first acquired pathological expertise at the Institute of Clinical Pathology in Freiburg for her future work as head of the ophthalmic pathology laboratory. Then, with Thomas Reinhard taking office in 2003, the magic threshold of 1000 specimens was finally exceeded (Fig. 2), their number now increasing by an average of 90 per year, more than doubling to 2046 samples in 2015. In summary, the annual number of specimens has increased significantly across the various medical directors’ tenures (*p* < .001), as shown in Fig. 3.

Witschel, Loeffler and Auw-Haedrich became full members of the European Ophthalmic Pathology Society (EOPS). Auw-Haedrich habilitated in 2006 and in the same year had the ophthalmic pathology laboratory certified according to DIN EN ISO 9001; this certification is still valid today. An ophthalmopathological case conference was held and continues to be conducted on a weekly basis with internal and external participants, in which interesting or unclear cases are presented and discussed. It provides clinicopathological correlation to improve the attendees’ clinical and histopathological diagnostic skills and serves as further training for medical colleagues interested in ophthalmic pathology. Digital imaging was introduced where glass histological slides are scanned in a slide scanner producing high-resolution digital colour images that are equally advantageous for morphological analysis in diagnostics including internal or external consultations, in teaching and research. Our laboratory observes the requirements of Regulation (EU) 2017/746 of 5 April 2017 on In vitro Diagnostic Medical Devices (IVDR).

Alongside advancements in medical and surgical practices, the dedication to ophthalmic pathology and, ideally, the expertise of the various heads of the Freiburg Eye Center, a third crucial driver for the observed increase across the study period is the demographic growth in the city of Freiburg and its catchment area, as well as the gradual expansion of the latter. The frequency distribution of histological case numbers between 1945 and the mid-1990s appears to follow a similar shape to the population growth curve in the city of Freiburg (Fig. 2). The number of inhabitants climbed from 89,275 at the end of 1945 to 160,007 in 1967, to 183,979 in 1988, to 210,234 in 2002, finally reaching 226,393 in 2015 [39], to mention some of the key data along our timeline. In addition, with increasing life expectancy thanks to improved nutrition, education and healthcare, the population also faces a higher risk of developing tumours and other eye conditions we see at our hospital. As a consequence, additional medical staff had to be recruited to cope with the growing demand for surgical interventions obviously even surpassing the population growth, which could only be met thanks to Mackensen’s meanwhile well-established microsurgical techniques and follow-up examination of excised specimens at our ophthalmic pathology lab.

A fourth, albeit less pronounced, driver since the mid-1990s has been additional specimens from external submitters such as eye hospitals, pathology laboratories and practising ophthalmologists. These external samples contributed on average around 10% to the particularly strong increase in sample numbers since around 1998 (Fig. 4). Between 1994 and 2015, specimens were submitted for examination at our Specialised Ophthalmic Pathology Laboratory from 135 different locations throughout Germany, including some from Switzerland.

### Patient age and sex

The left-skewed bimodal frequency distributions of the number of specimens by recorded patient age at surgery (range: 0–108 years) for female, male and all patients combined (Fig. 5) show local maxima (minor modes) at age 2 (f) and age 1 (m, all) with a range of 0–5 years of age for which a total of 809 surgeries were recorded. Associated diagnoses are attributable largely to benign eyelid tumours (*n* = 302; 37%), 75% of which (28% of all surgeries) were dermoid cysts, a common benign tumour in children occurring predominantly below the lateral edge of the eyebrow; 12% were haemangioma, 8% molluscum contagiosum and 5% were other, rarer diagnoses. Enucleated eyeballs accounted for 19% (*n* = 153) after malignant tumours (44%) had been diagnosed, 94% of which (8% of all surgeries) were retinoblastoma usually diagnosed before the age of 2, congenital malformations (25%), trauma (10%), glaucoma (7%) and other causes for enucleation (14%). Chronical inflammation of the eyelid (*n* = 78; 10%), of which 74% were a chalazion, benign conjunctival tumours (*n* = 35; 4.3%), including 46% nevi, 23% cysts, 17% lipodermoids and 14% papillomas, chronical conjunctivitis (*n* = 20; 2.5%), comprising pyogenic or foreign body granulomas (40%), and 221 (27%) other diagnoses complete the diagnostic profile in children aged 0–5 years.

For the pronounced shoulder peak at 66 years in the frequency distribution of samples from female patients and for the less prominent occurrence at 67 years in male patients, the 20 most common diagnostic categories (“Top 20”) are listed in Tables 1 and 2, with ages 66 and 67 combined for each sex. The sequence of the most common diagnostic categories in females starts with benign eyelid tumour (23%), malignant AMP eyelid tumour (12%), inflammatory eyelid lesion (mostly chalazion) (7.4%) and normal eyelid tissue (mostly re-resection specimens) (6.3%), followed by corneal dystrophy (mostly Fuchs’ dystrophy except in 3 cases) (5.3%), pterygium and pinguecula (5.2%) and degenerative corneal changes (4.8%). These 7 diagnostic categories account for 64% of a total of 842 diagnoses in female patients. In male patients, the “Top 20” list of diagnostic categories is headed by benign eyelid tumour (22%), followed by malignant AMP eyelid tumour (11%), inflammatory eyelid lesion (mostly chalazion) (9.1%), pterygium and pinguecula (7.3%), normal eyelid tissue (mostly re-resection specimens) (5.5%), corneal dystrophy (all Fuchs’ dystrophy) (4.6%) and degenerative corneal changes (4.1%). Here again, these same 7 diagnostic categories represent 64% of the now 821 total diagnoses in male patients. We see no significant changes in these patterns as we move to neighbouring lower and higher ages, and there are no particular age-related features in the respective diagnostic spectrum. Therefore, we speculate that there may rather be social reasons for the observed (shoulder) peaks, such that predominantly female and male patients may have had postponable surgeries until they reached retirement or pensionable age, generally 65 years in Germany until 2012.

In the major modes at 72 (m) and 74 (f) years of age at surgery (Fig. 5), the most common topographical regions biopsied for histopathological examination were (m and f patients): eyelid (49% and 49%), cornea (17% and 19%) and conjunctiva (13% and 12%), followed by the less frequent eyeball (7.3% and 7.7%) and temporal artery (7.6% and 8.0%) (Fig. 6). This is consistent with observations at most sites regardless of age during the study period, while the relative frequency of surgical specimens taken from the temporal artery is twofold higher at ages 72 and 74 than their average proportion of 3.9% across all patient ages. Following Stålhammar et al. [4], we attribute the 2-year difference between the two main modes to the difference in life expectancy at age 72, which on average was 2.4 years (range: 2.2–2.6) higher for German women than for men during our study period (1945–2015) [40]. The 20 most common diagnostic categories (“Top 20”) at age 74 (f) and at age 72 (m) are shown in Tables 1 and 2. In females, the spectrum of the 7 most common diagnostic categories representing 67% of 505 diagnoses in total is similar to that found at ages 66 and 67 years. While the contribution of benign eyelid tumours decreased by 4%, that of malignant AMP eyelid tumours, for instance, has grown by 5%, corneal dystrophies (mostly Fuchs’ dystrophy) increased by 4.7% and the “Top 7” now include inflamed temporal artery (4.6%). In male patients, the 7 most frequent diagnoses contributing 63% of 430 diagnoses in total basically show the same pattern as found at ages 66 and 67, with little changes in the individual contributions. While benign eyelid tumours now contribute 5% less, the frequency of the other diagnostic categories has increased accordingly. As expected, inflamed temporal artery does not appear among the “Top 7” in males.

The shapes of the frequency distribution of specimen numbers by the Swedish female and male patient age at surgery (range: 0–104 years) published by Stålhammar et al. [4] show a remarkable agreement with our own age distributions (Fig. 5). Their main modes are shifted upwards by 2 years to age 76 for Swedish female patients, and to age 74 for Swedish male patients, likely due to a 2 years higher life expectancy of the Swedish compared to the German population [41]. The difference of 2 years between the two modal values is in line with our own results. The authors explain this observation by reference to the higher life expectancy of Swedish women (by 5 years) compared to men over their study period (1959 to 2021) [4]. Secondary mode values for both sexes are found at age 3; however, associated diagnoses (including retinoblastoma) are not detailed. Almost consistently across all ages the number of specimens excised from Swedish females is higher than that from males, while in our distributions this is only the case beyond the major mode values. Another frequency distribution of interest is that by age of US patients at surgery (range: 2–102 years; time period 1941–1995) by Spraul and Grossniklaus [17], which has a similar shape to our distributions as well (Fig. 5). Their major mode is at 74 years, as found in our female patients. Their secondary frequency peak is at an age of 6 years and is more pronounced, which is 3 to 4 years higher than the minor modes observed in [4] and found in our own data. The mean age of patients with retinoblastoma was 1.84 years at the time of enucleation [17]. More detailed information on diagnoses leading to surgery within the age range 2–10 years was not provided.

### Topographical regions

We conducted a retrospective review of ocular pathology reports, including clinical data on all surgical specimens examined and archived at the Specialised Ophthalmic Pathology Laboratory at the Eye Center at Medical Center, University of Freiburg. This review covers the period since the lab resumed operation in December 1944, after the hospital’s complete destruction. Leading histological diagnoses were classified according to five main topographical regions and a summarised group “other location”. Although we used a similar procedure to comparable studies, overlaps between groups cannot be completely ruled out.

Upon examining at the changes in relative frequency of histological samples from main topographical areas across the observation period (Fig. 7, Table 3), two notable features are immediately apparent: a fourfold statistically significant increase in the percentage of eyelid lesions in each time interval from 13% in 1945–1954 to 54% in 2005–2015 (*p* < .001), and a strong statistically significant 38-fold decrease in the proportion of eyeball specimens (enucleations) from 61% in 1945–1954 to 1.6% in 2005–2015 (*p* < .001). If we consider individual years, however, these changes are even more pronounced. The relative frequency of eyelid lesions climbed 28-fold from 2.1% in 1945 to 59% in 2010, and that of enucleations dropped 64-fold from 77% in 1945 to 1.2% in 2015 (not explicitly shown). At the same time, the annual number of eyelid lesions increased more than 1000-fold from 1 in 1945 to 1,043 in 2010, while the number of eyeballs increased 3.4-fold from 36 in 1945 to 121 in 1981, before they decreased fivefold to 24 in 2015 (not explicitly shown). Median values of annual specimen numbers within each interval increased significantly 111-fold across our study period, from 8 eyelid samples p.a. in 1945–1954 to 888 p.a. in 2005–2015 (*p* < .001). Medians of enucleated eyeballs increased significantly 2.4-fold from 39 p.a. in 1945–1954 to 92 p.a. in 1975–1974, then they significantly decreased fourfold to 24 p.a. in 2005–2015 (p < .001).

Across our study period, we found significant differences in the annual numbers of eyelid, corneal and conjunctival specimens between the four medical directors’ tenures: gr1 (1945–1967, Wegner), gr2 (1968–1987, Mackensen), gr3 (1988–2002, Witschel) and gr4 (2003–2015, Reinhard) (*p* < .001). For *eyelid* and *conjunctival* samples, the mean ranks of the following pairs were significantly different: gr1-gr2 (each *p* < .001), gr1-gr3 (each *p* < .001), gr1-gr4 (each *p* < .001), gr2-gr3 (*p* = .015 and .018), gr2-gr4 (each *p* < .001), and for *corneal* samples: gr1-gr2 (*p* < .001), gr1-gr3 (*p* < .001), gr1-gr4 (*p* < 0.001), gr2-gr4 (*p* < .001), gr3-gr4 (*p* = .035). In other words, eyelid, conjunctival and corneal specimens increased significantly from Wegner (gr1) to Mackensen (gr2), then there was a significant increase in eyelid and conjunctival samples from Mackensen (gr2) to Witschel (gr3). Finally, we see that corneal samples increased significantly from Witschel (gr3) to Reinhard (gr4). The apparent “non-significant” increase in eyelid and conjunctival specimens from gr3 to gr4 was likely due to the number of samples submitted annually within Reinhard’s tenure (gr4) extending over a wide range (eyelid: 489–1043 specimens p.a., conjunctiva: 192–309 specimens p.a.).

#### Eyelid

Initially, there was a clear predominance of tumours (83%) compared to other diagnostic categories, slightly in favour of malignant (42%) over benign ones (41%) (Table 5), while this ratio changed towards the end of our study period in favour of benign tumours. This may have been due to prioritisation following the Second World War, when only limited numbers of both surgeries and histological examinations could be performed. As a result, surgery on clinically suspected malignant tumours was likely given higher priority and these were removed more frequently followed by histological examination.

The general increase in eyelid specimens received for histological examination was partly due to the growing population and an expansion of our hospital’s catchment area and thus to a general increase in surgery numbers. Cosmetic reasons became a focus of attention, for example when considering the removal of a benign tumour or a chalazion in the case of inflammation (Table 5). In addition, there has been increasing pressure to economise during the past decades leading to more cost-effective thinking on the part of ophthalmologists in private practice. As a consequence, patients were sent to the nearest eye clinic more frequently to undergo surgeries (e.g., excision of malignant tumours) which tend to be not sufficiently remunerated and are therefore performed less and less frequently by private practices. Thirdly, Table 5 shows that “other eyelid diagnoses” increased over time and since the 1990s accounted for a significant proportion of eyelid lesions, at 21% in 1995–2004 and up to 26% in 2005–2015. This primarily refers to re-resection after previous removal of a tumour, but not in healthy tissue according to histological examination; in some cases, multiple re-resections were required.

#### Cornea

The proportion of corneal lesions in each time interval rose significantly by more than fivefold during the study period, from 4.4% in 1945–1954 to 22% in 2005–2015 (*p* < .001), while their numbers also increased 135-fold from 27 to 3,656 (Table 3). Median values of corneal specimen numbers per year within each interval increased significantly 120-fold across our study period, from 2.5 corneal samples p.a. in 1945–1954 to 302 annually in 2005–2015 (*p* < .001). Corneal surgery had already become more frequent after Mackensen took over directorship, from a mean of 4 ± 2.3 cases annually in 1945–1967 to an average of 51 each year in 1968–1987. When Witschel assumed management, these numbers rose to 104 in 2002, but it was only under Reinhard’s directorship, who had made corneal treatment and surgery one of his specialities, that the number of corneal specimens submitted to histological analysis soared from 161 in 2003 to 536 in 2015 (not shown). Corneal dystrophy contributed 27% to all corneal surgical specimens in 2005–2015 (Table 8), most of which (92%) were diagnosed with Fuchs’ endothelial dystrophy (Table 9). We found a statistically significant increase in the mean relative frequency of Fuchs’ dystrophies among the corneal lesions between neighbouring intervals, from 14.1% in 1995–2004 to 29.6% in 2005–2015 is (*p* < .021). This effect is likely due to the introduction of DMEK (Descemet membrane endothelial keratoplasty) in 2010, allowing indications to be established much earlier and resulting in more surgeries to be performed. The number of DMEKs has risen steadily from 23 specimens in 2011 to 139 in 2013 to 299 in 2015. Conversely, the relative contributions of macular, lattice and granular dystrophy declined from 7.9%, 13% and 11% respectively in the years 1965–1974 when they were first diagnosed, to 1.4%, 1.7% and 1.2% in 2005–2015. Their ratio only slightly changed from 1.0: 1.6: 1.4 in 1965–1974 to 1.0: 1.2: 0.86 in 2005–2015 (Table 9).

A slight decrease of keratoconus within the corneal specimens from the 1980s onwards (Table 8) is most likely explained by advances in treatment. On the one hand, an improvement in the fitting of dimensionally rigid contact lenses could have prolonged the period of acceptable visual quality and delayed the decision for penetrating keratoplasty. We see a more pronounced decrease from 18 to 10% within the last two observation intervals. This correlates with the introduction of cross-linking in corneal collagens as a new therapy for keratoconus in 1997 [42], as suggested by Lang et al. [43]. In addition, the observed decline in keratoconus may also be due to an increase in the relative frequency of corneal dystrophies, which almost doubled from 17 to 27% over the last two decades, especially after the introduction of DMEK in 2011 as a new and faster treatment for Fuchs dystrophy, while at the same time the absolute number of keratoconus cases increased from 217 in 1995–2004 to 446 in 2005–2015. The overall ratio between samples from male (*n* = 676) and female patients (*n* = 288) was m/f = 2.3:1 (Table 10).

The sharp and significant increase in the number of corneal lesions since the beginning of Reinhard’s tenure (*p* < 0.001) is also noticeable in corneal diagnostic sub-categories (Table 8): Corneal inflammation caused by herpes or bacteria, more frequent graft failure associated with an increased number of corneal transplants, and scar tissue after inflammation or injury. Bullous keratopathy without previous cataract surgery increased from 23 (2.8%) of 817 corneal samples in 1985–1994 to 288 (6.6%) of 4,382 corneal specimens in 2005–2015, while BK after surgery declined from 114 cases (14%) in 1985–1994 to 79 cases (1.8%) in 2005–2015. By the end of the 1990s, the ratio of proportions shifted significantly in favour of bullous keratopathy without prior phacoemulsification (not shown). This is most likely due to technological improvements in surgical equipment and advancements in surgical techniques, which lead to fewer complications such as a bullous keratopathy. In Freiburg, phacoemulsification became a routine in the late 1980s [44]; the same was reported of Sweden for the beginning of the 1990s [45]. Improvement of phacoemulsification techniques in the mid-1990 s [46] such as smaller incisions and improvements to the phacoemulsification machines resulted in less intraoperative endothelial cell loss [47, 48].

In the case of corneal ulcers (contributing 5.6% in 2005–2015), we observe an increase in samples from elderly patients in particular; problems in general care or in eye care are likely to be a factor here, especially in the presence of risk factors such as eyelid malposition or neurotrophic keratopathy. Malignant corneal tumours play only a very minor role, with just 8 samples (0.7%) of 1,216 corneal specimens arriving in our laboratory for histological examination in 1995–2004, the highest number in a 10-year interval during the entire study period (Table 8).

#### Conjunctiva

The relative frequency of conjunctival lesions within each interval increased significantly across our study period (*p* < .001) by doubling from 7.8% in 1945–1954 to 16% in 2005–2015 (Table 3). Remarkable was also the increase in the numbers of conjunctival specimens, from 48 in 1945–1954 to 2,675 in 2005–2015. Similar to corneal lesions, the number of histologically examined conjunctival specimens increased from 8 ± 5.1 (mean ± *SD*) each year between 1945 and 1967 to an average of 38 lesions annually within Mackensen’s directorship. Numbers grew from 49 in 1988 to 203 in 2002 during Witschel’s tenure until their further increase under Reinhard’s medical directorship to 309 in 2015 (not shown). Median values of conjunctival specimen numbers per year within each interval increased significantly 44-fold across our study period, from 5 conjunctival samples p.a. in 1945–1954 to 220 p.a. in 2005–2015 (*p* < .001).

While degenerative conjunctival changes contributed just 13% (*n* = 58) of 431 specimens in 1975–1984, their proportion reached 45% (*n* = 1,271) of 2,849 conjunctival lesions in 2005–2015 (Table 13). These were predominantly pterygia, the pathogenesis of which is highly correlated with UV exposure. The rising frequency of corresponding histological samples in our laboratory during the last decades could be explained by increasing rates of immigration from countries with a dry climate and/or close to the equator as well as by the general increase in UV exposure in our latitudes.

A growing population and catchment area translate into more patients with benign conjunctival tumours which are essentially rarer than eyelid tumours. Their contribution to conjunctival lesions declined from 51% (*n* = 218) of 431 conjunctival specimens in 1975–1984 to 21% (*n* = 609) of 2,849 specimens in the last interval 2005–2015 (Table 13). A growth in specimen numbers associated with the overall increase in histological samples was also observed for conjunctival inflammation, with not much of a change in relative frequency within conjunctival diagnostic categories (16% in 1975–1985; 13% in 2005–2015). Our data show that malignant conjunctival tumours occur even less frequently than benign tumours. However, these UV light-associated malignant tumours were diagnosed more often since the mid-1990 s. The ratio between benign and malignant conjunctival tumours of 5.3:1 in 1965–1974 and 4.8:1 in 1985–1994 dropped to only 2.2:1 in 1995–2004 and even further to 1.7:1 in 2005–2015 (Table 13). In other words, whereas in the 1970s and 1980s only one in five conjunctival tumours was of a malignant nature, this occurred in less than one in two conjunctival tumours by the end of our study period.

#### Eyeball

While their number initially increased from 374 in the first decade to 945 in 1975–1984, it subsequently declined to 274 in 2005–2015. The rather dramatic and statistically significant decrease in the proportion of enucleations in each interval, from 61% to 1.6% across our study period (*p* < 0.001), should be viewed in the context of the overall 27-fold increase in surgical specimens received between 1945 and 2015, primarily associated with the other main topographical regions (Table 3). The aforementioned increase in the frequency of enucleations observed until the fourth decade was mainly due to trauma (30%) often leading to proliferative vitreoretinopathy, malignant tumour (19%), vascular disease (11%), glaucoma (5.6%), bulbar inflammation (5.6%) and postoperative complication (4.9%) (Table 16).

Mackensen had introduced vitrectomy in the early 1970s, but at that time this technique still caused considerable damage especially to the retina. It was not until the mid-1980 s that the gentler 3-way vitrectomy was introduced, resulting in notably fewer injuries and explaining the decline in trauma-caused enucleations from around 1985 (Table 16). The decrease in number and frequency of enucleations is further associated with improved conservative treatment options for malignant tumours using radiotherapy and for glaucoma by lowering the intraocular pressure (IOP), primarily through surgical means but also by medication. In the first four decades, vascular disease became more common as prosperity gradually increased since the post-war era and patients adopted unhealthier diets, making them more susceptible to obesity and hyperlipidaemia. This caused circulatory disorders, which may ultimately have led to the loss of vision and of the eye. Treatment of vascular diseases in general has improved since the mid-1980 s, and laser therapy has been introduced to promote retinal oxygen supply. The number and relative frequency of bulbar inflammations hardly change across the study period (Table 16). These are mainly post-operative infections or inflammations of unknown origin. The use of improved antibiotics and treatment by vitrectomy to rapidly eliminate bacteria, in combination with intra/post-operative antibiotics, may have prevented an increase in these cases despite the rising number of surgical interventions.

The number of postoperative complications as underlying cause for enucleation increased only slightly during the study period. These include retinal detachments requiring multiple operations, and expulsive haemorrhages following intraocular surgery. It is very likely that improved retinal surgery techniques as in trauma-associated vitrectomy, have significantly reduced the complication rate here as well. In cataract surgery, reducing the incision size led to the prevention of intra/postoperative negative pressure and, as a result, expulsive haemorrhages. Retinal detachments as a cause of enucleation initially increased with the growing population and catchment area, reaching a fraction of 8% in the interval 1995–2004, albeit at a relatively low level. However, advancements in surgical treatment options, particularly vitrectomy, have since resulted in preservation of the eyeball in most cases (Table 16). While numbers and relative frequency of phthisis specimens generally remained at very low levels throughout the study period, we cannot offer an explanation for the – compared to the other intervals (mean: 6.4 cases) – sixfold elevated number of 38 cases (8.7%) of 438 enucleations observed within the 1994–2005 interval.

#### Temporal artery

Diagnostic methods for pathologies of the temporal arteries were not introduced until the 1960s, which is why they did not appear until 1959 in the second decade of our study (not shown). Within the main topographic regions, their share rose noticeably in the third decade, reaching a peak of 15% in 1985 (not shown; average: 7.9% in 1975–84, 8% in 1985–1994) and significantly declining (*p* < .001) to 2.2% towards the end of our study period (Table 3). From 64 cases in 1965 to 1974, the numbers increased to 389 lesions in 1975–1984 (Table 18). Median values of annual numbers of temporal artery biopsies within each interval increased significantly fivefold from 8 specimens p.a. in 1965–1974 to 40 p.a. in 1985–1994, followed by a significant decrease to 21.5 lesions p.a. in 1995–2004 and again a slight rise to 35 biopsies p.a. in 2005–2015 (*p* < .001).

It is not uncommon that the original suspicion of inflammation turns out to be a degenerative change or a normal finding. However, since only individual vessel sections can be affected, a negative biopsy result does not rule out the disease with certainty. In the years 1965–1974, only 22% of suspected diagnoses turned out to be actual arteritis temporalis, while we found degenerative changes (mostly intimal hyperplasia) in 44% and normal findings in 34% of the 64 cases. Similarly in 1975–1984: While numbers had grown sixfold to 389 specimens, only 18% of the suspected diagnoses turned out to be an inflammation, 56% were found to be degenerative changes and 26% of the cases revealed normal findings. In the following decade 1985–1994, however, the suspected diagnoses were remarkably often confirmed histologically, in 56% of 451 cases (Table 18). With introduction of pre-operative diagnostics using ultrasound in the mid-1990 s (today: colour coded duplex sonography and, if necessary, MRI), the number of temporal artery excisions decreased to 235 in 1995–2004, of which 49% were normal findings, 26% degenerative changes and 25% actually confirmed the suspected diagnosis. In 2005–2015, finally, the diagnosis “arteritis temporalis” was made in only 7% of 377 biopsies, while degenerative changes were present in 48% of the cases and 45% were normal findings.

### Comparison with other studies

The studies by Rohrbach et al. [16] and by Spraul and Grossniklaus [17] are particularly useful for comparison with the relative frequency of our own surgical specimens associated with a histological diagnosis and originating from different topographies. Both cover a broader spectrum of locations, although sample numbers and/or time periods may differ to some extent (Table 19). It is important to note that [16, 17] included temporal artery in their combined group “others”, and that in [17], eyeballs were not evaluated as a single location only but individual diagnoses were assigned to the respective region such as cornea, lens, vitreous, retina or optic nerve.

**Table 19:** Comparison of topographical areas

Author	Rohrbach et al. [16]						Spraul and Grossniklaus [17]	Current study
Region	Tübingen						Atlanta	Freiburg
Time period	1900	1920	1940	1960	1980	1990	1941–1995	1945–2015
Number	*n* = 98	*n* = 87	*n* = 239	*n* = 230	*n* = 656	*n* = 525	*n* = 24,444	*n* = 39,256
Eyelid	5%	17%	12%	34%	29%	45%	8%	50%
Cornea	0%	1%	3%	16%	23%	22%	37%	16%
Conjunctiva	3%	1%	4%	12%	10%	16%	7%	14%
Eyeball	51%	69%	37%	24%	20%	9%	^1)^	9.1%
Temporal artery	^2)^	^2)^	^2)^	^2)^	^2)^	^2)^	^2)^	3.9%
Orbit	1%	0%	3%	3%	1%	1%	2%	1.4%
Lens	^3)^	^3)^	^3)^	^3)^	^3)^	^3)^	15%	0.9%
Vitreous	^3)^	^3)^	^3)^	^3)^	^3)^	^3)^	11%	0.5%
Retina	^3)^	^3)^	^3)^	^3)^	^3)^	^3)^	7%	0.3%
Choroidea	^3)^	^3)^	^3)^	^3)^	^3)^	^3)^	9%	0.1%
Other location	38% ^4)^	11% ^4)^	40% ^4)^	10% ^4)^	18% ^4)^	8% ^4)^	3%	3.5% ^5)^

Relative frequencies of specimens from various topographical areas during the observation period (1945–2015) and comparison with one other study. Percentages may not total 100 due to rounding

^1)^ Eyeballs were not evaluated as a single location; individual diagnoses were assigned to the respective localisations (cornea, lens, vitreous, retina, choroidea) [17]

^2)^ Included in “other location” [16, 17]

^3)^ Not specified separately

^4)^ Includes intraocular tissue (1900: 16%, 1920: 3%, 1940: 21%, 1960: 4%, 1980: 13%, 1990: 4%), lacrimal duct (1900: 8%, 1920: 6%, 1940: 14%, 1960: 1%, 1980: 1%, 1990: 1%), temporal artery biopsies, gangliectomies (1900) and other unclassifiable and lost tissue samples (1900: 15%, 1920: 2%, 1940: 5%, 1960: 5%, 1980: 4%, 1990: 3%) [16]

^5)^ Does not include orbit, lens, vitreous, retina, choroidea

Our most common topographical areas were eyelid (50%), cornea (16%) and conjunctiva (14%), followed by eyeball (9.1%) and temporal artery (3.8%). Our combined “other location” group (3.5%) is now composed differently than originally stated in Table 3 and does not include orbit (1.4%), lens (0.9%), vitreous (0.5%), retina (0.3%) and choroidea (0.1%) for this comparison (Table 19). Rohrbach et al. [16], whose University Eye Hospital Tübingen is located just 106 km (66 miles) northeast of Freiburg, report strikingly similar relative frequency data for 512 specimens examined in 1990: eyelid (45%), cornea (22%) and conjunctiva (16%), followed by eyeball (9%), the group “others” including temporal artery (3%), and orbit (1%). However, looking further back in time, the sequence of the main regions changed in terms of their relative frequency, and the proportion of enucleated eyeballs has been way more prominent at the beginning of their study period: 51% in 1900, 69% in 1920, 37% in 1940, 24% in 1960 and 20% in 1980 (Table 19). This corresponds to the significant decrease observed in eyeball specimens submitted to our own lab: 61% in 1945–1954, 48% in 1955–1964, 22% in 1965–1974, 19% in 1975–1984 and 11% in 1985–1994 (Table 3). A statistically significant increase that we observed in the relative frequency of histologically examined eyelid specimens is reported by Rohrbach et al. [16] as well: 5% in 1900, 17% in 1920, 12% in 1940, 34% in 1960, 29% in 1980 and 45% in 1990 (Table 19). This series roughly corresponds with our own findings: 13% in 1945–1954, 23% in 1955–1964, 36% in 1965–1974, 45% in 1975–1984 and 51% in 1985–1994 (Table 3). Probable reasons for both observed changes have already been discussed. Rohrbach et al. [16] attribute the pronounced increase in examined eyelid specimens primarily to more frequent excision of benign, non-inflammatory and inflammatory changes (chalazion). The authors also rightly argue that in past decades, injuries and glaucoma used to dominate as reasons for enucleation, whereas around 1990 it was tumours, closely followed by traumata [16].

Spraul and Grossniklaus [17] describe a somewhat different picture at their ophthalmic pathology laboratory in Atlanta, GA, USA, with the following sequence of topographical areas in terms of their relative frequency from the time period 1941–1995: cornea (37%), lens (15%), vitreous (11%), choroidea (9%), eyelid (8%), conjunctiva (7%), retina (7%) and orbit (2%) (Table 19). On the one hand, in Spraul and Grossniklaus’ study [17], “eyeball” was not considered a separate entity in the overall distribution of locations throughout their study, but diagnoses were assigned to individual topographies. Therefore, the proportions of cornea, lens, vitreous body, retina and choroid were considerable, with the latter four alone accounting for 42%. However, in selected time periods within their study, eyeballs contributed 86.1% in 1941–1947, 61.2% in 1970, and 8.1% in 1995. The latter proportion compares well to our own results in 1985–1994 (11%) (Table 3) and with those reported by Rohrbach et al. [16] for 1990 (9%) (Table 19). Conversely, the low percentage of eyelids (8%) in [17] seems striking compared to our data (50%) and that of Rohrbach et al. [16] (45% in 1990). Reasons for this may be found in differences in the clinical procedures: in Atlanta, eyelid specimens were submitted to dermatopathology until 1989 [49], while in Freiburg, every eyelid sample, including every chalazion, has been and still is examined histologically so as not to overlook a sebaceous gland carcinoma, which explains the high number of eyelid samples. Just a few lens and vitreous specimens were histologically examined in Freiburg over time, whereas in Atlanta, this was the most common specimen processed histologically in 1995 [17]. Similarities between the three study periods can be found in changes in the relative frequency of corneal and conjunctival histological samples. Rohrbach et al. [16] report a proportion of 3% corneal samples in 1940 and 22% in 1990 (Table 19): in Spraul and Grossniklaus [17], this percentage was 0.5% in 1941–1947 and 21.2% in 1995, while we found 4.4% in 1945–1954 and 13% in 1985–1994 (Table 3). The contribution of conjunctival samples in Tübingen was 4% in 1940 and 16% in 1990 [16], in Atlanta it was 3.8% in 1941–1947 and 6.9% in 1995 [17], while of all samples examined histologically in Freiburg conjunctival specimens accounted for 7.8% in 1945–1954 and 10% in 1985–1994 (Table 3). Ongoing research projects and newly introduced or alternative surgical methods could also explain some of the observed differences.

#### Eyelid

The relative frequency of eyelid lesions with associated diagnostic categories from our archive was compared to previous results from five other studies conducted in Tübingen, Atlanta, Iowa City, Baltimore and Philadelphia [16–19, 21], chronologically arranged in Table 20 according to the beginning of their respective time interval. Diagnostic patterns were similar in each study population, benign tumour generally being the most common category, followed by inflammation, malignant tumour, and a combined group “other eyelid diagnosis”. One exception was inflammation being the leading diagnostic category (34%) in [17], although immediately followed by benign tumour (33%). Benign tumours comprised 37% of all histologically examined eyelid lesions in our study, compared with a range of 33–54% in four of the other studies and even 68% in the case of Welch and Duke [19]. We found that surgical specimens with inflammation and those with a malignant tumour accounted for 23% and 20% respectively, while ranges of 17–34% and 15–24% are reported in [16–19, 21].

**Table 20:** Comparison of various eyelid lesions

Author	Rohrbach et al. [16]	Aurora and Blodi [18]	Spraul and Grossniklaus [17]	Welch and Duke [19]	Tesluk [21]	Current study
Region	Tübingen	Iowa City	Atlanta	Baltimore	Philadelphia	Freiburg
Time period	1900–1990^a^	1932–1969	1941–1995	1952–1956	1980–1982	1945–2015
Number	*n* = 553	*n* = 892	*n* = 1,021	*n* = 617	*n* = 720	*n* = 21,764
Benign tumour	46%	54%	33%	68%	49%	37%
Inflammation	34%	22%	34%	17%	23%	23%
Malignant tumour	16%	24%	17%	15%	19%	20%
Other eyelid diagnosis	3.4%	-	16%	-	9%	19%^b^

Relative frequencies of various eyelid lesions with associated diagnostic categories and comparison with five other studies. Percentages may not total 100 due to rounding

^a^Specimens collected and diagnosed in 1900, 1920, 1940, 1960, 1980 and 1990 [16]

^b^For individual sub-categories see Table 7

Tables 21 and 22 compare the relative frequencies of benign and malignant eye tumours from our archive with the results of six [17–19, 21–23] and eight [16–23] previously published studies, respectively, suitable for this purpose. For better comparability of benign tumours, diagnostic categories were reviewed in six of the studies and, in some cases, regrouped [17–19, 21–23]. Based on the data of Spraul and Grossniklaus [17], we summarised squamous cell papilloma, seborrheic keratosis, benign keratosis, hidrocystoma, verruca, keratoacanthoma, pseudoepitheliomatous hyperplasia and epidermal inclusion cyst as *epithelial* tumour. Nevi were grouped as *melanocytic* tumour, hemangioma as *mesenchymal* tumour, and dermoid cyst as *choristoma*. From Deprez and Uffer`s study [22], we have grouped squamous cell papilloma, seborrheic keratosis, inverted follicular keratosis, pseudocarcinomatous hyperplasia, inverted follicular keratosis, keratoacanthoma, acanthoma, epidermal cysts and various benign tumours of the skin appendages into *epithelial* tumour. Melanocytic nevi were grouped as *melanocytic* tumour, vascular tumours and lipomas as *mesenchymal* tumour. Neurofibroma, neurinoma and neuroma were combined as *neuronal* tumour, and xanthelasma, xanthogranuloma, fibrous histiocytoma and other skin appendage tumours grouped as “other benign eyelid tumour”. Diagnostic categories reported by Domingo et al. [23] were regrouped in a similar fashion. Epidermoid cyst, squamous papilloma, seborrheic keratosis, benign sweat gland and hair follicle tumour were grouped as *epithelial* tumour. Melanocytic nevi were grouped as *melanocytic* tumour, and neurofibroma and neurinoma as *neuronal* tumour. Hemangioma, lipoma and lymphangioma were summarised in *mesenchymal* tumour, lymphoid hyperplasia to lymphocytic tumour. Xanthelasma and xanthogranuloma formed the combined group “other benign eyelid tumour”. Results of the studies by Aurora and Blodi [18], Welch and Duke [19] and by Tesluk [21] were restructured in a similar way. For comparison of the relative frequency of specimens diagnosed with a malignant tumour in eight previous studies with our database, results were taken directly from the respective publications [16–23]. For Rohrbach et al. [16], we summarised the data from the individual time periods given. Relevant details were indicated in the respective footnotes, especially regarding “other malignant eyelid tumour”.

**Table 21:** Comparison of benign eyelid tumours

Author	Aurora and Blodi [18]	Spraul and Grossniklaus [17]	Welch and Duke [19]	Tesluk [21]	Deprez and Uffer [22]	Domingo et al. [23]	Current study
Region	Iowa City	Atlanta	Baltimore	Philadelphia	Lausanne	Manila	Freiburg
Time period	1932–1969	1941 - 1995 ^1)^	1952–1956	1980 - 1982	1989–2007	2003–2012	1945–2015
Number	*n* = 471	*n* = 363	*n* = 387	*n* = 319	*n* = 4,404	*n* = 344	*n* = 8,131
Epithelial	68%	74%	61%	81%	71%	61%	73%
Melanocytic	17%	23%	23%	9.0%	19%	20%	15%
Mesenchymal	6.0%	2%	4.0%	-	2.0%	4.7%	4.3%
Choristoma	-	1.7%	6.7%	3.1%	0.7%	^2)^	1.7%
Neuronal	1.3%	-	-	-	0.4%	7.0%	0.4%
Lymphocytic	-	-	-	1.9%	^2)^	2.0%	0.1%
Other benign eyelid tumour	6.6%	-	5.0%	4.7%	7.2%	6.1%	0.8%

Relative frequencies of benign tumours of the eyelid grouped according to their cellular origin and comparison with six other studies. Percentages may not total 100 due to rounding

^1)^Specimens collected and diagnosed in 1941–1945, in 1970 and in 1995 [17]

^2)^Not specified separately

**Table 22:** Comparison of malignant eyelid tumours

Author	Aurora, Blodi [18]	Rohrbach et al. [16]	Spraul, Grossniklaus [17]	Welch, Duke [19]	Font et al. [20]	Tesluk [21]	Deprez, Uffer [22]	Domingo et al. [23]	Current study
Region	Iowa	Tübingen	Atlanta	Baltimore	Pasadena	Philadelphia	Lausanne	Manila	Freiburg
Time period	1932–1969	1940–1990 ^1)^	1941–1995	1952–1956	1970–2000	1980–1982	1989–2007	2003–2012	1945–2015
Number	*n* = 214	*n* = 91	*n* = 172	*n* = 88	*n* = 594	*n* = 125	*n* = 1,096	*n* = 170	*n* = 4,396
Basal cell carcinoma (AMP)	80.4%	68%	59.9%	77%	69%	82.4%	70%	30.6%	86%
Squamous cell carcinoma (EMP)	7.0%	25%	18.6%	19%	5%	2.4%	6.1%	17.1%	6.0%
Sebaceous gland carcinoma (EMP)	3.2%	^2)^	15.1%	1%	14%	6.4%	2.6%	30.6%	1.7%
Lymphoma (EMP)	1.8%	-	1.2%	-	3%	2.4%	-	6.5%	1.1%
Metastasis (EMP)	1.4%	^2)^	1.2%	-	1%	-	0.4%	1.2%	0.3%
Merkel cell carcinoma (EMP)	-	-	-	-	< 1%	-	0.4%	-	0.1%
Other malignant eyelid tumour	6.0% ^3)^	6.6% ^4)^	4.1% ^5)^	2.2% ^6)^	8%	6.2% ^7)^	20% ^8)^	13.6% ^9)^	4.4%

Relative frequencies of malignant tumours of the eyelid with associated diagnoses and comparison with eight other studies. Percentages may not total 100 due to rounding

^1)^ Specimens collected and diagnosed in 1940, 1960, 1980, 1990 [16]

^2)^ Not specified separately, included in “other malignant lid tumour”

^3)^ Includes melanoma (5.1%), Bowen’s disease (0.5%) and adenocarcinoma of gland of Moll (0.5%) [18]

^4)^ Includes sebaceous gland carcinoma, malignant melanoma, metastasis [16]

^5)^ Includes melanoma (2.9%) and carcinoma, poorly differentiated (1.2%) [17]

^6)^ Includes carcinoma in situ [19]

^7)^ Includes malignant melanoma (5.4%) and carcinoma in situ (0.8%) [21]

^8)^ Includes actinic keratosis (18%), squamous cell carcinoma in situ (1.3%), lentigo maligna (0.5%), lentigo maligna melanoma (0.2%) and Kaposi sarcoma 0.1%) [22]

^9)^ Includes melanoma (11.8%), malignant pilar structure tumours (1.2%), mucoepidermoid carcinoma (0.6%) [23]

When we now compare the relative frequency of our benign eyelid tumours with those from the literature [17–19, 21–23] (Table 21), a rather consistent picture emerges: most were epithelial tumours (73%), which is well in line with the range of 61–81% found in six previous studies. In descending order follow tumours of melanocytic (15%) and mesenchymal origin (4.3%). These findings also correspond to data from the literature (9–23% and 2–6%), as do our results for choristomas (1.7%) and neuronal (0.4%) and lymphocytic tumours (0.1%). 60% of choristomas (dermoid cysts), which account for an unusually high proportion of 6.7% of benign tumours in Baltimore [19], occurred in the first decade of life, suggesting a referral bias. Noteworthy is the high relative frequency of 7.0% neuronal eyelid tumours (neurofibromas) in young patients (average age: 18 years) reported by Domingo et al. [23] for their Philippine study population, whereas in Iowa City it is lower at 1.3% [18], and only 0.4% in Lausanne [22] and in our study.

In Table 22, the majority of our malignant tumours were basal cell carcinomas accounting for 86% of the cases, which is above the range found in most of the other studies (59.9–82.4%) [16–22]; as the only exception, Domingo et al. [23] report just 30.6%. This low proportion of basal cell carcinomas may be explained at least in part by the high percentage of sebaceous gland carcinomas (30.6%) in Manila [23]. This in turn is likely due to the fact that Asians are more susceptible to sebaceous gland carcinoma, with reported relative frequencies of 3–6% of malignant tumours in Western series, but 27–37% in Eastern series [22]. The more frequent occurrence in Atlanta (15.1%) [17] and as found by Font et al. [20] in Pasadena, California (14%) compared to our own findings (1.7%) and to that in other studies (1.0–6.4%) [18, 19, 21, 22] could also be due to a higher proportion of individuals of Asian origin in their patient populations. Another considerable contribution to malignant eyelid tumours reducing the percentage of basal cell carcinomas in the Philippine study population [23] is that of squamous cell carcinomas (17.1%) (Table 22), which are associated with higher age and UV exposure. Domingo et al.’s [23] findings are in line with the results of Rohrbach et al. [16] in Tübingen (25%), Spraul and Grossniklaus [17] in Atlanta (18.6%) and Welch and Duke [19] in Baltimore (19%). However, they stand in contrast to our own data (6%) and those of Aurora and Blodi [18] in Iowa City (7.0%), Font et al. [20] in Pasadena (5%), Tesluk [21] in Philadelphia (2.4%) and Deprez and Uffer [22] in Lausanne, Switzerland (6.1%). There are indications that the relative frequency of squamous cell carcinomas of the eyelid, similar to sebaceous gland carcinomas, is also increased in Asians [22, 50]. In addition to a possible referral pattern, a certain percentage of patients of Asian origin could be at least a partial explanation for the similarly high frequencies reported for the Atlanta [17] and Baltimore [19] study populations. However, it does not explain the high rate of occurrence in the predominantly Caucasian study population observed by Rohrbach et al. [16] in Tübingen. Other, yet infrequent malignant eyelid tumours in our study were lymphomas (1.1%), metastases (0.3%) and Merkel cell carcinomas (0.1%), ranging in a comparable order of magnitude to the results of six of the eight other studies [17, 18, 20–23].

#### Cornea

Table 23 shows the relative frequency of various corneal lesions with associated diagnostic categories during our study period, compared to results from four chronologically arranged previous studies from Tübingen, Atlanta, Australia and the United States [16, 17, 24, 25]. We only compared our results with studies in which diagnoses led to a surgical procedure during which samples were obtained for histological examination. The higher number of corneal samples in the Australian Corneal Graft Registry [24] is due to the fact that these are nationwide overview data from 1985–2020 of cases resulting in keratoplasty. Indications for grafts for all types of keratoplasty were analysed and regrouped for comparison where appropriate, as shown in Table 23.

**Table 23:** Comparison of various corneal lesions

Author	Rohrbach et al. [16]	Spraul and Grossniklaus [17]	Keane et al. [24]	Musch et al. [25]	Current study
Region	Tübingen	Atlanta	Australia	USA	Freiburg
Time period	1900–1990 ^1)^	1941–1995	1985–2020	2001–2007	1945–2015
Total number	*n* = 310	*n* = 8,039 ^2)^	*n* = 40,774	n/a	*n* = 7,319
Dystrophy	*n* = 22 ^3)^(7%)	*n* = 1,033 (14%)	*n* = 7,635 (19%)^5)^	*n* = 1,330^4)^n/a	*n* = 1,580 (22%)
* Fuchs’* ^6)^	^7)^	88%	94%	68%	86%
* Macular* ^6)^	^7)^	1.7%	0.8%	0.4%	3.0%
* Lattice* ^6)^	^7)^	2.8%	0.8%	1.9%	2.7%
* Granular* ^6)^	^7)^	1.5%	1.0%	1.5%	2.6%
* Other corneal dystrophy* ^6)^	^7)^	6.1%	2.4%	28%	5.3%
Keratoconus	*n* = 76 (25%)	*n* = 794 (11%)	*n* = 9,752^8)^(24%)	^9)^	*n* = 975 (13%)
Inflammation	^7)^	*n* = 1,389 ^10)^(19%)	*n* = 2,177^8)^(5.3%)	^9)^	*n* = 832 (11%)
Transplant failure	^7)^	*n* = 1,150 (16%)	*n* = 9,977^5)^(25%)	^9)^	*n* = 806 (11%)
Scar tissue	^7)^	*n* = 1,140 (15%)	*n* = 444^8)^(1.1%)	^9)^	*n* = 730 (10%)
Bullous keratopathy	*n* = 64 ^11)^(21%)	*n* = 1,267 (17%)	*n* = 7,161 ^12)^(18%)	^9)^	*n* = 713 (9.7%)
Malignant tumour	^7)^	*n* = 16 (0.2%)	*n* = 56 ^13)^(0.1%)	^9)^	*n* = 30 (0.4%)
Other corneal diagnosis	*n* = 148 (48%)	*n* = 1,250 (17%)	*n* = 3,572^5)^(8.8%)	^9)^	1,653 ^14)^(23%)

Numbers and relative frequencies of various corneal lesions during the observation period (1945–2015) and comparison with four other studies. Percentages may not total 100 due to rounding

^1)^ Specimens collected and diagnosed in 1900, 1920, 1940, 1960, 1980 and 1990 [16]

^2)^ Number of diagnoses; number of cases: *n* = 7,322; originating from corneal excisions and enucleations [17]

^3)^ Without Fuchs’ endothelial dystrophy [16]

^4)^ Percentages calculated from Table 3 in [25] including only cases requiring keratoplasty, this work includes corneal dystrophies only and no other corneal diagnoses

^5)^ Data extracted from lists in [24] of indications for graft of perforating keratoplasty (PK), DSAEK (Descemet’s stripping automated endothelial keratoplasty), DMEK (Descemet’s membrane endothelial keratoplasty), DALK (Deep anterior lamellar keratoplasty) and traditional lamellar keratoplasty

^6)^ Proportions given as percentage of dystrophy

^7)^ Not specified separately

^8)^ Data extracted from lists in [24] of indications for graft of perforating keratoplasty (PK), DSAEK, DALK and traditional lamellar keratoplasty

^9)^ Only corneal dystrophies are included in [25]

^10^^)^ Includes ulcer [17]

^11^^)^ Referred to as primary and secondary endothelial decompensation in [16]

^12^^)^ Data extracted from lists in [24] of indications for graft of perforating keratoplasty (PK), DSAEK and DMEK

^13^^)^ Data extracted from lists in [24] of indications for graft of perforating keratoplasty (PK), DSAEK and traditional lamellar keratoplasty

^14^^)^ Includes ulcer (*n* = 411, 5.6%); for other pathologies (*n* = 1,242, 17%) see Table 11

In Freiburg, corneal dystrophies accounted for the largest proportion of corneal lesions (22%), with Fuchs’ endothelial dystrophy being the most common dystrophy (86%), as expected. This is consistent with the results of Spraul and Grossniklaus [17] (Atlanta), Keane et al. [24] (Australia) and Musch et al. [25] (USA), although in the latter study Fuchs’ dystrophy only accounts for 68% of all dystrophies. This observed discrepancy might be due to the inclusion of clinical diagnoses in Musch et al.’s study (2001–2007) [25], while our results are based on histological diagnoses only. Another, perhaps even more likely explanation is the increasing use of the DMEK procedure after its introduction in 2008 by Melles [51], resulting in a faster surgical procedure and visual rehabilitation compared to perforating keratoplasty (PKP). In our ophthalmic pathology laboratory, we have seen a steady increase in Descemet’s membranes from 23 in 2011 to 139 in 2013 to 299 specimens in 2015, contributing to an increase in Fuchs’ dystrophy diagnoses. The comparably low proportion of dystrophy in corneal lesions of just 7% reported by Rohrbach et al. [16] (Tübingen) does not include Fuchs’ endothelial dystrophy (Table 23).

Corneal dystrophies that follow Fuchs’ dystrophy in frequency, albeit in small numbers, are macular dystrophy with 48 cases (3.0%), lattice dystrophy with 42 cases (2.7%) and granular dystrophy with 41 cases (2.6%). A similar distribution was published by Spraul and Grossniklaus [17] who reported 29 cases of lattice dystrophy (2.8%), 18 cases of macular dystrophy (1.7%) and 16 cases of granular dystrophy (1.5%) out of 1,033 corneal dystrophies requiring PKP. Based on the pattern of inheritance, it would be expected that autosomal recessive macular dystrophy is less common than autosomal dominant lattice and granular dystrophy, as shown in [25] and, to some extent, in [24]. Musch et al. [25] analysed records of almost 8 million enrolees in a national managed-care network throughout the United States who had an eye care visit in 2001–2007. Out of 1,330 corneal dystrophy cases requiring PKP, the authors found 5 enrolees with macular dystrophy (0.4%), 25 with lattice corneal dystrophy (1.9%), and 20 with granular corneal dystrophy (1.5%). In contrast, our findings could indicate a noticeably increased proportion of macular dystrophy (3.0%) in the Freiburg area (Table 23).

With few exceptions, the results for the remaining types of corneal lesions convey a fairly consistent picture. The contribution of keratoconus specimens to corneal lesions was 13% in our study and 11% in [17], approximately half of the keratoconus proportions reported in [16] (25%) and in [24] (24%). This could reflect differences in the relative frequency during the various study periods, as the number of keratoconus cases both in Freiburg (*n* = 975) and in Atlanta (*n* = 794) is at least ten times lower than in Australia (*n* = 9,752). However, the prevalence of keratoconus is more likely to vary between different ethnic groups, with higher rates in Asian and Middle Eastern than in Caucasian populations [52]. It can be assumed that the studies listed in Table 23 primarily include Caucasians and comparable preoperative treatment standards, so that the observed differences between Atlanta [17] and Freiburg compared to Australia [24] and especially Tübingen [16], which is located not far from Freiburg, cannot be satisfactorily explained from this perspective.

The percentage of corneal inflammation is only 5.3% in Keane et al.’s results [24] compared to 11% in our study and 19% in that of Spraul and Grossniklaus [17]. The latter’s relatively high percentage is likely due to the fact that corneal findings from enucleated or eviscerated eyes were included in addition to keratoplasty findings [17]. Of their 5,843 corneal buttons, only 7.8% were diagnosed with keratitis (not separately shown in Table 23), which, like the mere 5.3% keratitis cases in [24], is even lower than our own results (11%). This is consistent with the findings of Matthaei et al. [53], who reported keratitis as an indication for keratoplasty in 13.2% of cases in Europe, 9.1% in North America and 5.1% in Australia.

Transplant failures contributed 11% to corneal lesions in our study but their share is higher at 16% in Atlanta [17] and at 25% in Australia [24]. It could be surmised that our lower percentage is due to more intensive post-operative anti-inflammatory measures such as systemic immunosuppression in high-risk keratoplasties and consistent long-term low-dose topical steroids where there is a higher risk of rejection. The higher total number of keratoplasties results in a higher number of graft failures and might explain the higher overall rate in [24].

While we found a proportion of 10% of corneal scar tissue and [17] reported 15%, its relative frequency was only 1.1% in [24] (Table 23). Scars are sequelae of traumata or keratitis or corneal ulcers. Only those causing visual disturbance due to central opacification or (irregular) astigmatism require keratoplasty. It is unclear why the relative proportion in [24] is considerably lower in comparison to [17] and to our own findings.

The percentage of bullous keratopathy in surgical corneal samples was only 9.7% in our study, but 17% in Atlanta [17], 18% in Australia [24] and 21% in Tübingen [16]. The higher percentage found by Rohrbach et al. [16] could be due to the observation period being in the pre-phacoemulsification era before 1991, when higher rates of endothelial decompensation occurred following cataract surgery. Spraul and Grossniklaus’ study period ended in 1995 [17], when phacoemulsification was still developing towards smaller incisions and improved phacoemulsification machines, which might explain their higher rate compared to ours. This argument, however, does not explain the proportion of 18% in Keane et al. [24], whose study period was 1985–2020.

With 0.2%, 0.1% and 0.4%, respectively, malignant tumour samples in [17, 24] and in our study were equally rare contributors to corneal lesions (Table 23). Corneal tumours are generally rare, especially primary ones. Most cases are secondary involvement of conjunctival tumours [54].

#### Conjunctiva

The relative frequency of conjunctival lesions with associated diagnostic categories from our archive is compared with two previous studies from Baltimore [26] and Atlanta [17] in Table 24. While conjunctival degeneration was our leading diagnostic category with 36%, followed by benign tumour (26%), inflammation (16%), malignant tumour (11%) and a combined group “other conjunctival diagnosis” (10%), the sequences reported by Grossniklaus et al. [26] and by Spraul and Grossniklaus [17] with regard to their relative frequency show slightly different patterns. The proportions of benign tumour being the leading conjunctival lesion in [26] at 28% and in [17] at 26% show the best agreement between the three studies. We have already explained the high proportion of degeneration in our archive as a possible consequence of increased immigration from countries with dry climates and the general increase in UV exposure in our latitudes.

**Table 24:** Comparison of various conjunctival lesions

Author	Grossniklaus et al. [26]	Spraul and Grossniklaus [17]	Current study
Region	Baltimore	Atlanta	Freiburg
Time period	1923–1984	1941–1995	1945–2015
Number	*n* = 2,455	*n* = 1,617	*n* = 5,963
Degeneration	23%	20%	36%
Benign tumour	28%	26%	26%
Inflammation	16%	27%	16%
Malignant tumour	19%	21%	11%
Other conjunctival diagnosis	14%	8%	10%^a^

Relative frequencies of various conjunctival lesions with associated diagnostic categories and comparison with two other studies. Percentages may not total 100 due to rounding

^a^For individual sub-categories see Table 15

The proportion of surgical specimens histologically diagnosed with conjunctival inflammation in our study (16%) is in perfect agreement with the findings in Baltimore [26] (16%), while their percentage in Atlanta [17] is considerably higher at 27%. This might be caused by inclusion of conjunctival scrapings in [17], which are not mentioned in [26] and are not included in our conjunctival cases.

Malignant, predominantly UV-associated epithelial [55] and melanocytic tumours [56] (Table 25) comprised 19% of conjunctival lesions in Baltimore [26] and 21% in Atlanta [17], but just 11% at our Eye Center in Freiburg (Table 24). Located at a latitude of 48° N, Freiburg is at a greater distance to the equator than Baltimore at 39.3° N or Atlanta at 33.8° N; the population in the Freiburg area might therefore be less UV-exposed.

**Table 25:** Comparison of conjunctival tumours

Author	Grossniklaus et al. [26]	Spraul and Grossniklaus [17]	Shields et al. [27]	Domingo et al. [23]	Current study
Region	Baltimore	Atlanta	Philadelphia	Manila	Freiburg
Time period	1923–1984	1941–1995	1974–2015	2003–2012	1945–2015
*Benign tumours*	*n* = 690	*n* = 420	*n* = 2,022	*n* = 141 ^1)^	*n* = 1,578
Melanocytic	31%	34%	68%	24%	49%
Epithelial	52%	47%	9.1%	40%	41%
Mesenchymal	3.8%	7.9%	11.2%	^2)^	4.4%
Choristoma	8.0%	7.6%	5.0%	21%	3.8%
Lymphocytic	4.3%	3.8%	5.3%	7.1%	1.1%
Other benign conjunctival tumour	1.4%	0.5%	1.8%	7.8%	0.3%
*Malignant EMP-tumours*	*n* = 207	*n* = 188	*n* = 1,482	*n* = 71	*n* = 313
Melanoma	33%	46%	41%	25%	45%
Lymphoma	18%	12%	24%	17%	25%
Squamous cell carcinoma	47%	41%	30%	56%	25%
Other EMP-tumour	1.4%	1%	5.6%	1.4%	4.5%
*Malignant AMP-tumours*	*n* = 252	*n* = 146 ^3)^	*n* = 914 ^3)^	*n* = 28	*n* = 353
Epithelial precancerous lesion	89%	86%	31%	93%	86%
Melanocytic precancerous lesion	8.3%	14%	68%	-	13%
Basal cell carcinoma	2.4%	0.7%	0.7%	7.1%	0.8%

Relative frequencies of conjunctival tumours with associated diagnoses during the observation period (1945–2015) and comparison with four other studies. Malignant tumours were classified based on existing metastatic potential (EMP), absent metastatic potential (AMP) and on their cell of origin. Percentages may not total 100 due to rounding

^1)^ We excluded pyogenic granuloma

^2)^ Not specified separately

^3)^ Not listed as such in the original work [17, 27]; tumours were regrouped as explained in the text

In Table 25, the relative frequency of conjunctival tumours with associated diagnoses from our archive is compared with the results of four other studies [17, 23, 26, 27]. For comparability, diagnostic categories were reviewed and regrouped in some cases. Spraul and Grossniklaus [17] and Grossniklaus et al. [26] categorised conjunctival specimens into different types of lesions, including inflammatory/infectious, acquired epithelial, degenerative, pigmented, acquired subepithelial, congenital and miscellaneous lesions. There was no primary subdivision into benign and malignant tumours, therefore we formed subgroups of the respective lesions from these studies [17, 23, 26, 27]. Papillomas, cysts, squamous metaplasia and oncocytomas were counted as *epithelial* tumours. Nevi, primary acquired melanoses without atypia, benign melanoses, melanosis oculi, and melanocytomas were combined as *melanocytic* tumours. Hemangiomas, lipomas, fibromas, hemangiopericytomas, myxomas, lymphangiectasias, and lymphangiomas were counted as *mesenchymal* tumours. Dermoids, epibulbar choristomas and dermoid cysts were grouped as *choristomas*. Lymphoid hyperplasia and one case with Kimura disease were included in *lymphocytic* tumours. For malignant tumours with metastatic potential, we counted *melanomas*, *lymphomas*, *squamous cell carcinomas* and *other EMP tumours,* namely malignant fibrous histiocytoma and Kaposi sarcoma, analogous to our own work. We further grouped dysplasia cases as *epithelial precancerous lesions.* Primary acquired melanoses with atypia were counted as *melanocytic precancers* and one *basal cell carcinoma* as malignant AMP-tumour.

The conjunctival tumours included in the study of Shields et al. [27] were also regrouped. We included neoplastic changes and cysts in benign *epithelial* tumours. Benign *melanocytic* tumours comprised nevi, racial melanosis and ocular melanocytosis. Benign *mesenchymal* tumours included benign vascular, fibrous, myxomatous and lipomatous tumours except orbital fat herniation. *Choristomas* did not require to be regrouped. Benign *lymphocytic* tumours included cases with reactive lymphoid hyperplasia. “Other benign conjunctival tumours” consisted of neural, xanthomatous, lacrimal gland tumours and secondary benign eyelid and lacrimal gland tumours. Malignant EMP-tumours consisted of *melanoma*, *lymphoma*, *squamous cell carcinoma* and other EMP-tumours combining eyelid sebaceous carcinoma, extraocular extensions, Kaposi sarcoma, metastases, leukemic lesions, Langerhans cell histiocytosis. In malignant AMP-tumours, conjunctival intraepithelial neoplasia, squamous metaplasia, acanthosis and actinic keratosis were combined as *epithelial precancerous lesions.* Primary acquired melanosis with atypia were counted as *melanocytic precancerous lesions*; further included were *basal cell carcinomas*. The benign and malignant conjunctival tumours included in the study of Domingo et al. [23] were grouped analogously.

Melanocytic tumour was the leading conjunctival diagnostic category accounting for 49% of benign conjunctival lesions in our study, followed by epithelial (41%) and mesenchymal tumours (4.4%), choristoma (3.8%), lymphocytic tumour (1.1%) and the small group “other benign conjunctival tumour” with 0.3% (Table 25). Our sequence differs from that in previous studies of Grossniklaus et al. [26] (Baltimore), Spraul and Grossniklaus [17] (Atlanta) and Domingo et al. [23] (Manila), who found epithelial tumour to be the most common benign tumour at 52%, 47% and 40% respectively. Conjunctival papillomas, but not conjunctival nevi, are associated with external factors such as HPV infection. Conjunctival papillomas are mostly associated with low-risk human papillomavirus (HPV) subtypes 6 and 11 [57]. Regional differences have been described for (high-risk) HPV [58], however, such differences may also exist for low-risk HPV with a higher prevalence in Atlanta [17], Baltimore [26] and Manila [23] compared to Freiburg, which would explain the lower relative frequency of benign epithelial conjunctival tumours in our study.

Shields et al. [27], on the other hand, found a high percentage of melanocytic tumours (68%) in Philadelphia, while epithelial tumours accounted for only 9.1%. At the same time, they report the highest proportion of mesenchymal tumours at 11.2%. Since no obvious risk factors for nevi and benign mesenchymal tumours are known, we rather explain the different distribution pattern of benign conjunctival tumours in [27] by a referral bias.

Domingo et al. [23] report a higher proportion of choristomas (21%) in their Philippine study population (2003–2012) compared to Baltimore (8.0%; 1923–1984) [26] and Atlanta (7.6%; 1941–1995) [17] (Table 25). This difference is even more pronounced when compared to Philadelphia (5.0%; 1974–2015) [27] and especially to Freiburg (3.8%; 1945–2015). This observation is most likely due to the high proportion of children and young adults in the Filipino population (2008: 44.2% aged 0–19 yrs), followed by the United States (2009: 27.2% aged 0–19 yrs) [59] and Germany (2009: 19.0% aged 0–19 yrs), resulting in larger numbers of patients with choristomas in the Philippines requiring surgical treatment. The decline in the relative frequency of choristomas in the USA in the sequence Baltimore, Atlanta and Philadelphia may be explained by a trend towards a smaller family size in recent decades.

Lymphocytic tumours accounted for 3.8% to 7.1% of benign conjunctival tumours in [17, 23, 26, 27], whereas in our own study they contributed only 1.1%. Benign lymphocytic tumours are assumed to result from a chronic inflammatory response to antigenic stimulation [60], while the specific cause is unclear. Therefore, we cannot explain their observed low relative frequency in our study.

A comparison of the distributions of malignant tumours with metastatic potential shows differences between the individual studies, as well (Table 25). The patterns found in Freiburg and Philadelphia [27] largely coincide with the leading diagnosis being melanoma at 45% and 41%, respectively, followed by lymphoma and squamous cell carcinoma. The most common EMP-tumour in Atlanta [17] was also melanoma (46%), followed by squamous cell carcinoma at 41% and the lowest proportion of lymphoma (12%) within our comparison. Squamous cell carcinoma is the most common EMP-tumour in Baltimore [26] and in Manila [23], accounting for 47% and 56%, respectively, while in [23] the relative frequency for both melanoma (25%) and lymphoma (17%) appears to be comparatively lower than in most of the previous studies. Ethnic differences in the prevalence of conjunctival melanoma have been reported [61], with highest prevalence in non-Hispanic Whites of 6.02/million and much lower prevalence in Asians of 0.38/million. This may offer an explanation for the lower relative frequency of conjunctival melanoma in [23] despite high UV exposure in Manila. Lymphoma, especially Non-Hodgkin’s Lymphoma, which accounts for the major proportion of conjunctival lymphomas, is more common in Caucasians compared to other ethnic groups [62]. This goes in line with the lower relative frequency of lymphomas in Domingo’s study of the Filipino population [23]. We hypothesise that a more heterogenous composition of the study populations in Atlanta and Baltimore compared to that in Freiburg may be partly responsible for the lower frequency of lymphoma seen in [17, 26] as well.

In Freiburg, Baltimore [26] and Atlanta [17], malignant tumours without metastatic potential show a similar distribution (Table 25). Epithelial precancerous lesions as most frequent diagnostic category contributes 86% to 89% to AMP-tumours, followed by melanocytic precancerous lesions at 13%, 8.3% and 14% respectively. In Manila, epithelial precancerous lesions contributed even 93% [23], while no melanocytic precancerous lesions were reported, which may be due to the low total number of AMP-tumours included in this study (*n* = 28). The high rate of epithelial precancerous lesions consisting of conjunctival intraepithelial neoplasia is consistent with the elevated rate of squamous cell carcinomas mentioned above. This could be linked especially to a high UV exposure near the equator. High-risk HPV does not seem to be an issue as a study upon head and neck squamous cell carcinomas (not of the conjunctiva) revealed a low HPV-prevalence in the Northwest Philippines [63]. On the other hand, a high rate of HPV in invasive cervical cancer was found in the same country [64]. It is therefore unclear if HPV plays a role in the relative high frequency of squamous cell carcinomas in Domingo’s study [23]. The lower prevalence of conjunctival melanoma for Asians mentioned above [61] might also be valid for melanoma precursor lesions which could explain the missing cases of melanocytic precancerous lesions in Domingo’s findings.

Particularly striking is the inverse ratio (31:68) of the relative frequencies of epithelial and melanocytic precancerous lesions in Philadelphia [27] (Table 25); but as mentioned above, a referral bias cannot be ruled out. The proportion of basal cell carcinoma in the Philippines [23] is considerably higher with 7.1% than in Atlanta [17], Baltimore [26] and Philadelphia [27], and in Freiburg, Germany. This may be due to the small total number of cases, as there were only 2 cases with a basal cell carcinoma among the 141 specimens.

#### Eyeball

The frequency of occurrence of leading histological diagnostic categories of eyeball lesions (enucleations) examined in our ophthalmic pathology lab in Freiburg is compared in Table 26 with six earlier studies from Atlanta, Copenhagen, Erlangen, Leeds, Iceland and Toronto [17, 28–32]. Information on special features or on the inclusion or exclusion of individual categories can be found in the footnotes to the table. To facilitate the comparison of study results from different time periods, we included findings from four individual time intervals in our study (1945–1954, 1985–1994, 1995–2004, 2005–2015), as well as data from our entire observation period (1945–2015).

**Table 26:** Comparison of various eyeball lesions

Author	Spraul and Grossniklaus [17]	Current study	Hansen et al. [28]	de Gottrau et al. [29]	Saeed et al. [30]	Current study	Geirsdottir et al. [31]	Spraul and Grossniklaus [17]	Current study	Chan et al. [32]	Current study	Current study
Region	Atlanta	Freiburg	Copenhagen	Erlangen	Leeds	Freiburg	Iceland	Atlanta	Freiburg	Toronto	Freiburg	Freiburg
Time period	1941–1947	1945–1954	1975–1996^1^^)^	1980–1990	1984–2003	1985–1994	1992–2004	1995	1995–2004	2004–2013	2005–2015	1945–2015
Number	*n* = 117	*n* = 374	*n* = 1,028 ^2)^	*n* = 1,146	*n* = 285	*n* = 624	*n* = 56	*n* = 104	*n* = 438	*n* = 713 ^2)^	*n* = 274	*n* = 3,555
Trauma	48.7%	28%	18%	37.4%	22%	26%	39%	33.7%	10%	16%	20%	25%
Malignant tumour	12.8%	14%	27%	19.6%	19%	18%	20%	30.8%	19%	36%	11%	19%
Glaucoma	10% ^3)^	14%	26%	3.2%	17%	8.2%	20%	2.9% ^3)^	8.4%	21%	1.4%	8.6%
Vascular disease	6.0%	1.6% ^4)^	-	16.2%	-	14% ^4)^	-	7.7%	10% ^4)^	-	14% ^4)^	8.4% ^4)^
Bulbar inflammation	10.3%	8.3%	16%	7.0%	11%	6.6%	16%	2.9%	6.2%	17%	11%	6.7%
Surgical disease	9.4% ^5)^	-	-	8.9%	-	-	-	12% ^5)^	-	-	-	-
Postoperative complication	^6)^	4.6%	2.9%	-	1.4%	6.3%	-	^6)^	14%	^6)^	17%	6.6%
Retinal detachment	^6)^	0.8%	4.6%	4.0%	9.5%	5.5%	-	^6)^	8.0%	3%	5.4%	4.1%
Phthisis	^6)^	2.9%		3.4% ^7)^	11% ^8)^	0.8%	-	^6)^	8.7%	^6)^	2.9%	2.3%
Other eyeball category	2.6%	25% ^9)^	4.5% ^10)^	1.4% ^11)^	9.1%	15% ^9)^	5.4% ^12)^	10.6	15% ^9)^	7%	17% ^9)^	19% ^9)^

Relative frequencies of leading histological diagnostic categories of eyeball lesions (enucleations) and comparison of current study results at various time intervals with six other studies. Percentages may not total 100 due to rounding

^1)^ Specimens collected and diagnosed in 1975–76, 1985–86, 1995–96; the total number of diagnostic categories in the various intervals is comparable, except for glaucoma and trauma, which decrease over the entire observation period [28]

^2)^ Includes eviscerations [28, 32]

^3)^ Secondary glaucoma [17]

^4)^ Excludes diabetes

^5)^ i.e. cataract, primary glaucoma, retinal detachment and various corneal diseases, resulting in enucleation [17]

^6)^ Not specified separately

^7)^ Includes atrophia and not classified phthisis [29]

^8)^ Includes atrophia (12.5%) [30]

^9)^ For individual sub-categories see Table 17

^10^^)^ Includes phthisis bulbi (59%) [28]

^11^^)^ Miscellaneous lesion [29]

^12^^)^ Unknown (33%), congenital (67%) [31]

With the exception of interval 1995–2004, trauma was the most common cause for enucleation in our study period, followed by malignant tumour, glaucoma, vascular disease or bulbar inflammation in varying proportions (Table 26). Most of the studies used for comparison show similar patterns. The low proportion of trauma-related enucleation (10%) found in Freiburg in 1995–2004 is associated with the introduction of 3-way vitrectomy [65] in the mid-1980s. In contrast, Spraul and Grossniklaus report a remarkably high proportion of trauma [17] due to penetration (42.2%), contusion (26.7%), rupture (17.0%), intraocular foreign body (9.6%) or perforation (3.0%). While trauma in Atlanta accounted for 48.7% between 1941 and 1947, it still contributed 33.7% in 1995, with an overall female-to-male ratio of 1:3.4 [17]. Elevated proportions of trauma as underlying cause for enucleation were also found by de Gottrau et al. [29] in Erlangen between 1980 and 1990 (37.4%) and by Geirsdottir et al. [31] in the Icelandic study population in 1992–2004 (39%). In summary, trauma-associated enucleations decrease from the 1940s to the 2000s both in [17] and in our study, which, as mentioned above, is probably linked to the progress of intraocular surgery, especially vitrectomy. There is no correlation between the rate of trauma-associated enucleations and the size of the respective city. This appears to be due more to location-specific factors such as occupational health and safety with regard to eye protection and crime prevention, which cannot be assessed in the context of this comparison.

Hansen et al. [28] found a high percentage of malignant tumours of 27% in their Copenhagen study population, compared to findings by de Gottrau et al. [29] in Erlangen (19.6%), Saeed et al. [30] in Leeds (19%) and our own data from the Freiburg region (18%), during comparable time ranges. Even more pronounced was the percentage of malignant tumours in Atlanta in 1995 (30.8%) [17] and in Toronto 2004–2013 (36%) [32]. We assume that referral patterns may have played a significant role, as ocular tumour cases were referred to specialized tertiary care centres for intraocular tumours, such as in Copenhagen [28], Atlanta [17] and Toronto [32].

Table 26 further shows differences in the relative frequency of glaucoma which is elevated at 17% to 26% in Leeds [30], Iceland [31], Toronto [32] and Copenhagen [28]. These higher proportions compared to results from Atlanta [17] and our study are most likely explained by the inclusion of cases with neovascularization glaucoma in [28, 30–32]. After exclusion of neovascularization glaucoma, the relative frequency in Iceland is 4% [31] and in Toronto 8.4% [32]. These results are now comparable to Erlangen (3.2% in 1980–1990) [29], Atlanta (10% in 1941–1947, 2.9% in 1995) [17] and Freiburg, where glaucoma occurred at 8.2% in 1985–1994, 8.4% in 1995–2004, 1.4% in 2005–2015 and at 8.6% across the entire study period. Especially since the mid-1980 s, the relatively low frequency of glaucoma-based enucleations has been associated with improved conservative and surgical treatment options as already discussed.

Vascular disease, where reported, occurred on a similar scale to ours: 6.0% in Atlanta in 1941–1947 [17] vs 1.6% in our study in 1945–1954, 16.2% in Erlangen in 1980–1990 [29] vs 14% in Freiburg in 1985–1994, or 7.7% in Atlanta in 1995 [17] vs 10% in our ophthalmic pathology lab in 1995–2004 (Table 26). The higher frequency of enucleation with vascular etiology found in Freiburg from the 1980s onwards most likely reflects the increased level of prosperity after the Second World War, with a higher frequency of diseases of civilization such as high blood pressure and elevated blood lipids. Improvements in therapy and nutrition may have led to a slight decrease in their relative frequency from the 1990s onwards. This is in line with the epidemiological development of cardiovascular diseases, with cardiovascular-related mortality decreasing by 60.2% and 45.9% in both Western Europe and North America between 1990 and 2022. Western Europe ranks 15th in age-standardized cardiovascular mortality in 1990 and 19th in 2022, while North America ranks 17th in both 1990 and 2022 [66].

Hansen et al. [28] report an elevated percentage of 16% bulbar inflammation leading to enucleation in Copenhagen (1975–1996), as do Geirsdottir et al. [31] for their Islandic study population (1992–2004), while Chan et al. [32] found 17% in Ontario (2004–2013) (Table 26). In the other three studies [17, 29, 30], as well as in our own, this proportion does not exceed 11% and may even be as low as 2.9%, as was found in Atlanta in 1995 [17]. These differences in relative frequency of bulbar inflammation as underlying cause of enucleation likely reflect treatment failures that may vary within this inhomogeneous group, probably consisting of different proportions of non-infectious and infectious cases. Regardless of therapeutic improvements in the administration of anti-inflammatory and antimicrobial agents, there remains a certain percentage of failures that eventually lead to enucleation.

In Freiburg, postoperative complications caused 6.6% of enucleations during the entire observation period, whereas this proportion may not always be included (separately) in other studies. Their increase to 14% in 1995–2004 and to 17% in 2005–2015 is mainly based on the generally observed decline in the number of enucleations, from 945 in 1975–1984 and 438 in 1995–2004 to only 274 in 2005–2015 (Table 16), reasons for which we have already discussed. In addition, the numbers of postoperative complications resulting in enucleation remained fairly constant since 1975–1984, despite a sharp rise in the overall number of surgeries, especially since 2002.

The relative occurrence of both retinal detachment (9.5%) and phthisis (11%) appears to be elevated in patients who underwent enucleation in Leeds in 1984–2003 [30], when compared with other studies from this time period [28, 29], including our own results from 1985–1994 (Table 26). However, Saeed et al.’s findings [30] are more consistent with our data from 1995–2004 (8.0% and 8.7%).

Overall time trends across the included studies [17, 28–32] cannot be derived due to the diversity of geographical regions and individual characteristics in the various hospitals, health care systems and study populations.

#### Temporal artery

Table 27 provides an overview of the relative frequency of temporal artery diagnostic categories during our study period compared to the results of five previous studies from Baltimore, USA [33], Sydney, Australia [34], Edmonton, Calgary, Quebec and Ottawa in Canada [35], Buenos Aires, Argentina [36] and Edirne, Turkey [37]. Special features or the inclusion or exclusion of individual categories are explained in the footnotes to the table. Our investigation period covers the individual time frames of the comparative studies, which in total extend from 1968 to 2016. At first glance, a fairly uniform picture emerges, while only in the study by McDonnell et al. [33] and in our work the data on “no inflammation” are further broken down.

**Table 27:** Comparison of temporal artery specimens

Author	McDonnell et al. [33]	Oh et al. [34]	Weis et al. [35]	De la Torre et al. [36]	Yuksel et al. [37]	Current study
Region	Baltimore	Sydney	Canada	Buenos Aires	Edirne	Freiburg
Time period	1968–1983	1992–2015	2005–2010	2005–2016	2011–2016	1945–2015
Number	*n* = 250	*n* = 538	*n* = 119	*n* = 63	*n* = 42	*n* = 1,517
No inflammation	83.2%	76.6%	76%	73%	81%	72%
Degenerative	74%	^1)^	^1)^	^1)^	^1)^	38%
Normal	6.4%	^1)^	^1)^	^1)^	^1)^	34%
Other	2.8% ^2)^	-	-	-	-	
Inflammation	12.8%	23.4%	24%	26.9%	19%	28%
Healed arteritis	4.0% ^3)^	-	-	-	-	

Relative frequencies of temporal artery diagnostic categories during the observation period (1945–2015) and comparison with five other studies. Percentages may not total 100 due to rounding

^1)^ Not specified

^2)^ Includes 4 biopsies containing segments of peripheral nerve, muscle, or vein without temporal artery; 2 biopsies interpreted as showing atypical focal injuries to the vessel wall and that could not be assigned to any other diagnostic category; and 1 biopsy interpreted as showing an apparently traumatic disruption of the vessel wall [33]

^3)^ Diffuse, marked intimal thickening; intimal and medial fibrosis, sometimes with vascularization; fragmentation, loss of internal elastic lamina; may have localized, full-thickness loss or fibrous replacement of media; adventitial scarring; foci of lymphocytes may be present [33]

Our most common diagnosis was the absence of inflammation in 72% of histological temporal artery specimens, with 38% of these showing degenerative changes and 34% were normal findings. McDonnell et al. [33] found no inflammation in 83.2% of temporal artery specimens, of which 74% were degenerative changes and only 6.4% were normal findings. In the studies from Australia, Canada, Argentina and Turkey, specimens without inflammation accounted for 73–81% of all temporal artery lesions [34–37]. Biopsies from temporal arteries diagnosed with inflammation represented the second largest proportion (19–28%) in most of the studies, with the exception of [33] where inflammation was clearly stated only in 12.8% of all cases submitted. In the same Baltimore study population [33], 4.0% of the cases were diagnosed with “healed arteritis temporalis” including secondary changes following arteritis, which may but need not show inflammatory infiltrates. Considering our results from the corresponding period of 1975–1984 with 18% inflammatory changes (Table 18), the sum of inflammatory cases (12.8%) and healed arteritis (4.0%) in [33] is quite comparable at 16.8%. As for the high percentage of biopsies lacking inflammatory changes in all of the above-mentioned studies, including our own, there is evidence that clinical giant cell arteritis with a negative temporal artery biopsy represent a true phenotypic variant and that negative biopsy results are not only explained by technical artefacts [67].

### Limitations and strengths

We have conducted a retrospective analysis of all histological specimens with corresponding records collected in our ophthalmic pathology laboratory’s archive from 1945 to 2015. Specimens included in this study were limited to tissue requiring ophthalmic surgical intervention. Changes in therapeutic standards, surgical techniques and capacities, the ophthalmologist’s decision to perform surgery, patient willingness to undergo an operation, decisions regarding submission for histopathological examination, changes in the geographic expanse of the hospital’s service area and demographical developments may be among the most important sources of bias and/or variation. These factors can limit the epidemiological significance of the frequency, prevalence and severity of various ocular diseases in the general population. Referral to a large tertiary eye care centre may increase the proportion of patients with rare causes of ocular disease, multiple diagnoses and diseases that do not respond to standard treatment. A large service area such as that of the Freiburg Eye Center can also mean that especially elderly patients living at a greater distance and those in the close vicinity may not be equally represented.

Nevertheless, our study utilises a large comprehensive set of histological slides and associated data of almost 40,000 specimens obtained over a period of 71 years, including information on the patient’s age and sex, the date of surgery and the histopathological diagnosis. Another strength of our study lies in the fact that the history of ophthalmology and ophthalmic pathology in Freiburg is well documented, so that observed characteristics and trends in the frequency of the various surgical specimens over time can be linked to historical events, novel surgical techniques and new treatment options. 22 previous studies with histological samples from various topographies served for comparison with our own results and discussion of similarities and differences. Our study data contribute to providing an overall picture of the type and relative frequency of ocular conditions leading to surgical excision of specimens with subsequent histopathological examination.

## Conclusions

Our review of ophthalmic pathology reports with clinical data of 39,256 histological specimens archived between 1945 and 2015 at the Specialised Ophthalmic Pathology Laboratory of the Eye Center at Medical Center, University of Freiburg reveals that most surgical specimens originated from the eyelid (50%), followed by cornea (16%), conjunctiva (14%), eyeball (9.1%), temporal artery (3.9%) and other locations (6.7%) comprising 16 less frequent topographical regions. An important factor at the beginning of the observation period was the proximity to the Second World War. The interval-based proportion of eyelid lesions increased statistically significantly (*p* < .001) and quadrupled from 13 to 54% over the 71 years of our study, while that of enucleations significantly decreased 38-fold from 61% to 1.6% (*p* < .001). The mean relative frequency of conjunctiva specimens doubled from 7.8% to 16% (*p* < .001) and that of excised corneal specimens climbed fivefold from 4.4% to 22% within the observation period (*p* < .001). Introduction of DMEK in 2010 led to a significant increase in the proportion of Fuchs’ dystrophies among the corneal lesions from 14.1% in 1995–2004 to 29.6% in 2005–2015 (*p* < .021). The mean percentage of temporal artery biopsies significantly decreased 3.6-fold from 7.9% in 1975–1984 to 2.2% in the last interval (*p* < .001).

At the same time, we observed a significant 111-fold increase in annual numbers of eyelid samples (*p* < .001), a 120-fold increase in corneal excisions (*p* < .001) and a 44-fold increase in conjunctival specimens (*p* < .001) across our study period. Annual numbers of enucleated eyeballs significantly rose 2.4-fold from 1945–1954 until 1975–1974, then significantly decreased fourfold until 2005–2015 (*p* < .001). Temporal artery biopsies significantly increased fivefold from 1965–1974 until 1985–1994, followed by a significant decrease in 1995–2004 and a slight rise again in 2005–2015 (*p* < .001). The total number of samples histologically examined each year increased significantly across the various medical directors’ tenures (indicated are medians): 78 samples p.a. (1945–1967, Wegner), 454 samples p.a. (1968–1987, Mackensen), 670 samples p.a. (1988–2002, Witschel) and 1,445 samples p.a. (2003–2015, Reinhard) (*p* < .001). Significant were also the increases in annual numbers of eyelid, corneal and conjunctival specimens between the four medical directors’ tenures (*p* < .001).

Observed changes in the frequency of various ocular and periocular specimens were linked to historical events, general developments in the population (increasing age, changes in diet, cosmetic reasons), new surgical techniques and treatment options and growing UV exposure. 22 previous studies involving ophthalmic pathology specimens from various topographical areas were used for comparison with our own findings. Similarities and differences were discussed and linked to the availability of (novel) surgical techniques, clinical procedures, the age-related or ethnic composition of the respective study populations, the geographical location or climatic influences.

The patient age at surgery was documented in 38,845 cases (99%). The shape of the bimodal frequency distributions of specimens by age of female, male and all patients combined was found to be similar in Freiburg to that recorded for the city of Atlanta, USA, and its catchment area between 1941 and 1995, and to a respective distribution for the entire Swedish population from 1959 to 2021.

We believe that the data from our study contribute to providing an overall picture of the nature and relative frequency of ocular and periocular conditions leading to surgical excision of specimens with subsequent histopathological examination. The remarkable increase in ophthalmic pathology samples observed since 1987 exceeding the demographic trend underlines the constantly growing importance and demand of the sub-speciality “ophthalmic pathology”. The clinically and surgically experienced ophthalmic pathologist usually recognises at first glance the histological structures present in a tissue section. Her or his comprehensive clinical and surgical ophthalmological expertise in the extraordinarily delicate and complex ocular and periocular anatomy, along with the wide range of anomalies and diseases of the eye and its adnexa, ensures the highest quality of ophthalmic pathology reports. Ideally, histopathological assessments should therefore be conducted by experienced ophthalmologists with expertise in both surgical procedures and pathology, or by experienced pathologists with expertise in ophthalmology, to ensure optimal patient-oriented care.

## Data Availability

The data that support the findings of this study are not openly available due to reasons of sensitivity and are available from the corresponding author upon reasonable request. Data are located in controlled access data storage at the Eye Center at Medical Center, University of Freiburg (Germany).

## Abbreviations

ACGR: Australian Corneal Graft Registry
AMP: Absent metastatic potential
ANOVA: Analysis of variance
BK: Bullous keratopathy
*χ*^2^: Pearson chi-squared value
CIN: Conjunctival intraepithelial neoplasia
*D*: Dip statistic
DALK: Deep anterior lamellar keratoplasty
DMEK: Descemet membrane endothelial keratoplasty
DSAEK: Descemet membrane endothelial keratoplasty
EMP: Existing metastatic potential
*η*^2^: Effect size, eta-squared value
*F*: F statistic
f: Female
gr: Group
HPV: Human papillomavirus
HSD: Honest Significant Difference
int: Interval
IOP: Intraocular pressure
IVDR: In vitro Diagnostic Medical Devices Regulation
m: Male
*Mdn*: Median
*p*: Probability
PKP: Perforating keratoplasty
*W*: Shapiro–Wilk test statistic
